# Nanoparticle-enriched mass spectrometry proteomics in British South Asians identifies links between genetic variants, plasma protein levels and disease risk

**DOI:** 10.1038/s41588-026-02697-6

**Published:** 2026-07-24

**Authors:** Maik Pietzner, Alice Williamson, Karen A. Hunt, Mine Koprulu, Leonhard Kohleick, Kamil Demircan, Genes & Health Research Team, Karen A. Hunt, David A. van Heel, Sarah Finer, Claudia Langenberg, Sarah Finer, Julia Carrasco Zanini, David A. van Heel, Claudia Langenberg

**Affiliations:** 1 Precision Healthcare University Research Institute, Queen Mary University of London, London, UK; 2 Computational Medicine, Berlin Institute of Health at Charité—Universitätsmedizin Berlin, Berlin, Germany; 3 Friede Springer Cardiovascular Prevention Center at Charité, Charité University Medicine Berlin, Berlin, Germany; 4 Blizard Institute, Queen Mary University of London, London, UK; 5 Wolfson Institute of Population Health, Queen Mary University of London, London, UK; 6 Max Planck Institute for Molecular Genetics, Berlin, Germany

**Keywords:** Population genetics, Genetics research

## Abstract

Understanding genetic variation associated with differences in plasma protein levels can elucidate human disease mechanisms. Here we demonstrate how untargeted nanoparticle-enriched mass spectrometry (MS)-based plasma proteomics delivers quantitatively and qualitatively different insights compared to two affinity-based assays in a sample of ~1,400 British South Asian individuals. We identify >1,200 significant locus–protein associations (*P* < 8.7 × 10^−12^; *n* = 895 *cis*-protein quantitative trait loci (pQTLs)), more than half of which have not been reported previously. Cross-platform comparison demonstrated that multiple platforms are required to capture the full spectrum of pQTLs of blood proteins. We combine proteogenomic results with evidence from multiple biological domains to suggest a potential role of 21 proteins in the pathology of 44 diseases, including a previously uncharacterized role of immunoglobulin λ variable 3-21 in the development of Graves’ disease. Our results demonstrate the potential of MS-based blood proteomics in non-European ancestries for pQTL discovery and the need to consolidate proteogenomic evidence to confidently assign proteins to disease pathology.

## Abstract (web summary)

This study reports genetic effects on mass spectrophotometry-based plasma proteomics in a cohort of ~1,400 British South Asians, accessing parts of the proteome missed by other proteomics platforms. The resulting protein quantitative trait loci are integrated with genome-wide association study data to find potential mechanisms of disease.

## Main

Proteins circulating in blood are under genetic control^1^, and linking protein quantitative trait loci (pQTLs) to complex diseases can inform drug target selection or drug repurposing in humans^2^. The most comprehensive studies so far used affinity-based methods, using either antibodies^3–6^ or single-stranded DNA aptamers^7–13^, to measure up to ~5,000 predefined proteins. However, between one-third^3^ and half^13^ of the assayed proteins could not be linked to variation near or within the corresponding protein-coding gene even in the studies of >30,000 participants. Mass spectrometry (MS)-based proteomic studies included 500 to 1,200 proteins at moderate sample size, identifying proteins and pQTLs not detected in larger affinity-based efforts^14–17^. However, the large dynamic range of the plasma proteome^18^ limited the coverage of low-abundant signaling proteins. Nanoparticle (NP)-based enrichment coupled with next-generation MS instruments can now identify >5,000 proteins circulating in blood at population scale^19,20^, providing unique opportunities for translational research.

Here we present a comprehensive MS-based plasma proteogenomic study of >5,700 protein groups robustly measured in >1,400 volunteers from the Genes & Health (G&H) cohort of adult British Bangladeshi and British Pakistani ancestry participants^21^. These communities are at disproportionally high risk for cardiometabolic and other diseases^21^, and have been historically underrepresented in genetic studies. High rates of autozygosity in this cohort further enable the detection of rare variants as homozygotes^22^, providing additional power for nonadditive genetic discovery.

## Results

We performed a comprehensive MS-based plasma proteomic study using the Seer Proteograph XT (referred to ‘Seer XT’ hereafter), identifying a total of 8,079 protein groups (Methods) detected at least once across 1,612 plasma samples of recalled volunteers of the G&H cohort of British Bangladeshi and Pakistani ancestry participants (*n* = 1,464, mean age = 43.4 years (12.2 s.d.), 55.9% females; mean body mass index = 27.7 kg m^−^^2^ (5.2 s.d.); 19.4% current smokers; Supplementary Table 1). This included 5,768 protein groups seen in ≥70% of samples, demonstrating the depth and coverage of the MS-based plasma proteome.

### MS-based plasma proteomics covers distinct fractions of the blood proteome

**Fig. 1:**
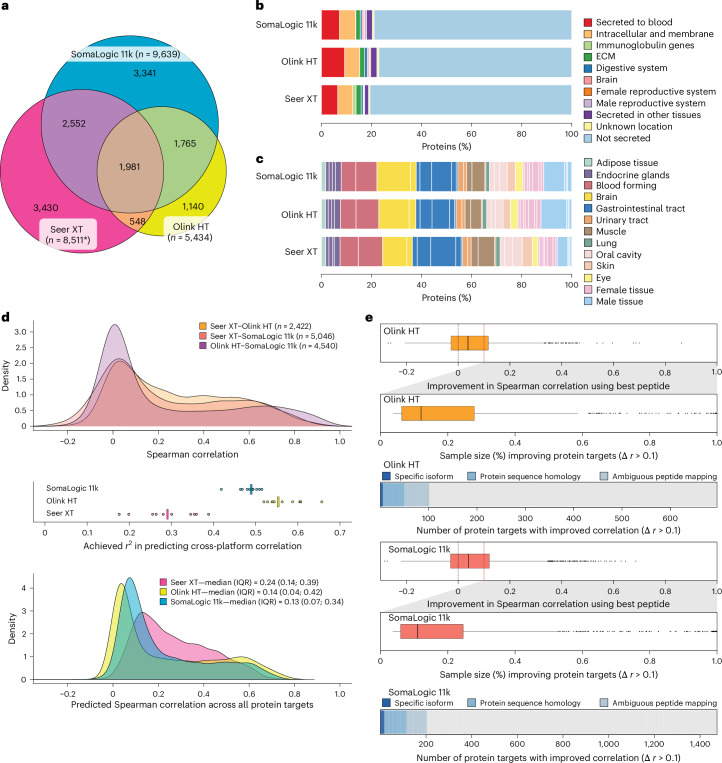
Quantitative and qualitative differences in MS and affinity-based plasma proteomics. **a**, Euler diagram showing the overlap of proteins captured by each platform (based on unique UniProt identifiers). An asterisk indicates some protein groups mapped to multiple UniProt identifiers. **b**, Assignment of secretome categories according to the Human Protein Atlas for each platform. **c**, Fraction of protein targets with evidence for enhanced production in certain tissues (separated by lines) based on mRNA expression patterns reported by the Human Protein Atlas. **d**, Top, distribution of Spearman correlation coefficients comparing measurements on the same samples for overlapping protein targets. Middle, achieved *r*^2^ in predicting the best correlation value for each platform based on technical and other characteristics of the platform listed. Each dot results from one cross-validation step, and the bar indicates the median performance. Bottom, the subsequently predicted correlation coefficients even for targets unique to each of the platforms. **e**, Comparing the best-correlating MS-based peptide instead of protein groups with matching measurements from affinity-based efforts (Olink HT, top; SomaLogic 11k, bottom). Box plots indicate the distribution of improvement (red line, Δ *r* > 0.1) in correlation and associated sample size for the respective peptide. Bar charts indicate potential explanations for improvements, if any. Box plots show median (centerline), IQR (box bounds) and 1.5× IQR (whiskers); points beyond whiskers are individual outliers. For Olink HT comparisons, *n* = 721 protein targets showed improvement in Spearman correlation of >0.1; for SomaLogic 11k, *n* = 1,401. The associated sample size for the best-correlating peptide varied per protein target and reflected the number of individuals with nonmissing measurements (range = 1–1,429 individuals; *n* = 1,396 individuals had measurements on all three platforms). Bar charts classify improvements by bioinformatic annotation; categories are mutually exclusive.

We identified more than 3,430 proteins (1,941 detected in >70%) in our NP-coupled MS-based assay that were not covered by the two currently most comprehensive affinity-based platforms, SomaLogic 11k (*n* = 4,533 proteins shared) and Olink HT (*n* = 2,529 proteins shared). This highlighted a substantial fraction of the circulating plasma proteome that is currently largely unexplored (Fig. 1a and Supplementary Tables 1–3). Notably, only a small fraction of proteins detected by MS, but also covered using other platforms, were predicted to be secreted into blood^23^ (Seer XT = 6.6%, SomaLogic 11k = 7.2%, Olink HT = 9.3%), providing evidence that most of the proteins readily detectable in blood originate from diverse sources and mechanisms (Fig. 1b).

**Extended Data Fig. 1:**
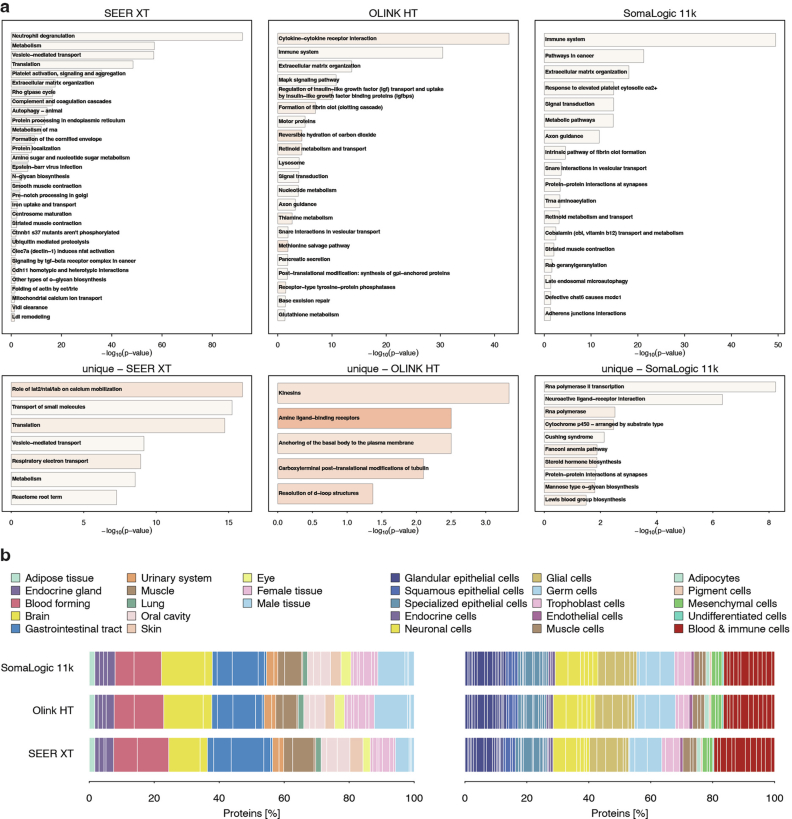
Cross-platform coverage of protein targets. **a**, Enriched pathways among proteins covered by each of the three platforms listed. We reduced the number of redundant pathways by selecting only those that share minimal overlap with the most significant ones. Color intensity is proportional to the fold enrichment of pathways. Top, pathways enriched among all proteins covered. Bottom, pathways enriched among protein targets uniquely covered by the platform listed. Pathway enrichment was tested using two-sided Fisher’s exact test with Benjamini–Hochberg FDR correction across all tested pathways. Only pathways with FDR < 5% are shown. The number of proteins per platform used as background: Seer XT, n = 8,511; Olink HT, n = 5,434; SomaLogic 11k, n = 9,639. **b**, Coverage of protein targets with evidence for enhanced production in tissues (left) or cell types (right) inferred from gene expression data based on bulk RNA sequencing in tissues or single-cell types. The underlying data were downloaded from the Human Protein Atlas^45^.

**Extended Data Fig. 2:**
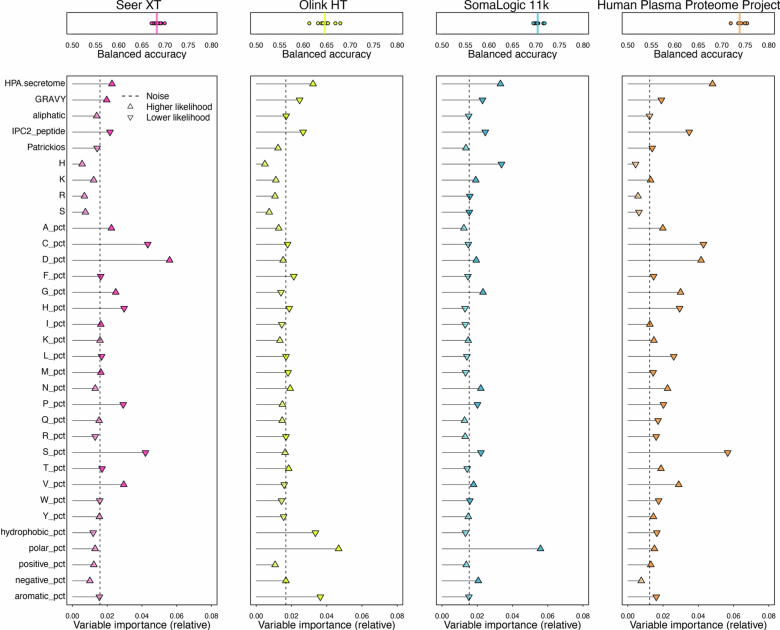
Results for machine learning models trained to predict assay coverage across platforms. The top layer contains, for each technology, the achieved balanced accuracy across ten outer folds of cross-validation. The corresponding panels below each show the median achieved variable importance for each feature selected at least once. The black vertical lines indicate noise levels as derived by the highest variable importance achieved by random variables used to monitor modeling. Letter codes represent proteinogenic amino acids. Pct = percentage.

We observed that the differential coverage across all three technologies translated into shared and distinct pathways and tissue/cell-type contributions enriched among proteins targeted by each platform (Fig. 1c and Extended Data Fig. 1). Proteins detected by MS showed the highest number of mutually exclusive pathways (Extended Data Fig. 1a and Supplementary Table 4) and were, for example, enriched for processes specific to blood cells, like neutrophil degranulation (odds ratio = 1.89; *P* < 9.7 × 10^−93^). In contrast, proteins captured by affinity-based platforms were most significantly enriched for unspecific immune signaling pathways (for example, SomaLogic 11k—odds ratio = 1.27; *P* < 3.6 × 10^−50^), while extracellular matrix (ECM) proteins were enriched across all platforms (Extended Data Fig. 1a). We corroborated these quantitative differences by profiling tissue or cell-type specificity using bulk tissue and single-cell mRNA expression data (Fig. 1c). For example, proteins likely specific to blood forming organs (9.8%, *n* = 832) and gastrointestinal tissues (11.5%, *n* = 982) were most prevalent among those measured with MS, whereas targeted affinity-based platforms showed higher fractions of proteins likely specific to the brain or sex-specific tissues. Physical properties of proteins partially explained differential platform coverage, with proteins captured by affinity reagents being enriched for high polar amino acid content (Extended Data Fig. 2 and Supplementary Note 1).

### Technical variation explains poor cross-platform agreement of protein measurements and enables prediction of concordance

**Extended Data Fig. 3:**
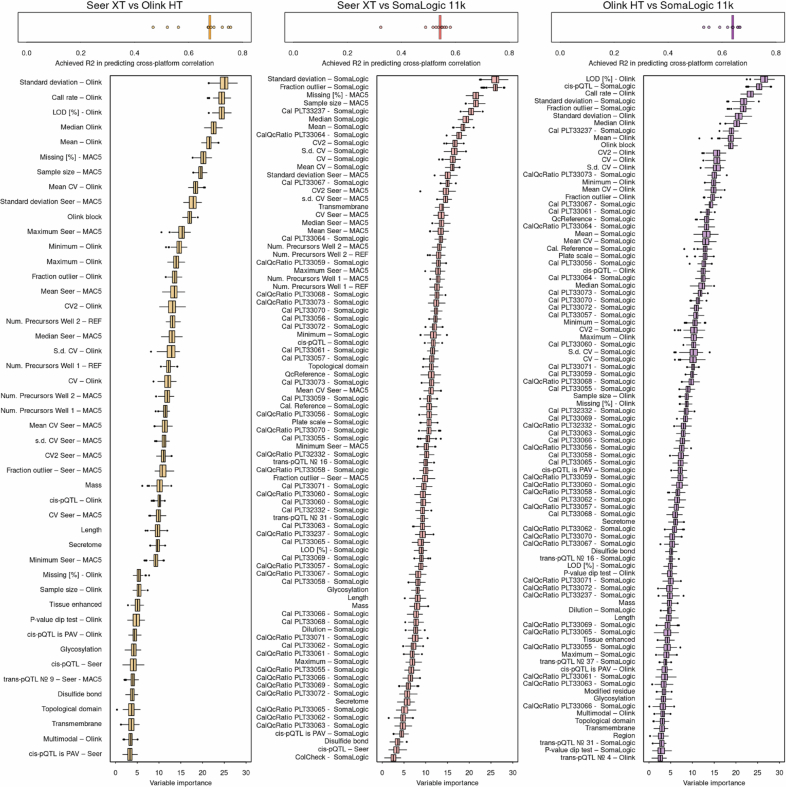
Features predicting cross-platform correlation of protein targets. Model performance (top) and associated feature importance (bottom) for three models (listed on top) to predict the Spearman correlation coefficients for overlapping protein targets. Achieved accuracy is based on a random forest classifier trained using 10-fold cross-validation. Feature importance was derived using the Boruta feature selection approach and only variables with confirmed significance (P < 0.05 following multiple testing correction) are shown. LOD = limit of detection; CV = coefficient of variation; pQTL = protein quantitative trait loci; PAV = protein altering variant.

**Extended Data Fig. 4:**
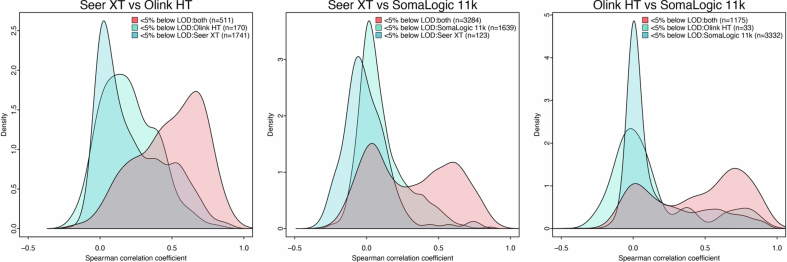
Cross-platform correlation gradients stratified by the estimated limit of detection (LOD) across assays. Densities are shown for proteins with <5% of values below LOD across both assays (pink); <5% in one (turquoise) or the other assays (blue; see legend). Numbers indicate protein targets used to compute densities.

Plasma proteomic platforms have been reported to only partially agree^13,19,24–26^, which we replicate here across 6,843 proteins overlapping across at least two platforms (Fig. 1d and Supplementary Tables 5–7). Assay sensitivity was among the most predictive characteristics when trying to explain assay concordance (Extended Data Fig. 3). Stratifying by the fraction of values below the detection limit of the assay (LOD) improved median correlation coefficients, for example, from 0.22 (interquartile range (IQR) = 0.05–0.48) considering all matching measurements to 0.51 (0.32–0.68) when considering 511 protein targets for which <5% measurements were below LOD for the MS-based assay and the Olink HT platform (Extended Data Fig. 4). These improvements were, however, less pronounced when comparing each platform to the SomaLogic 11k assay (Extended Data Fig. 4).

Generally, we observed that most of the (dis)agreement in measurements could be explained (54–67%) by technical factors and host genetic information (Extended Data Fig. 4 and Supplementary Table 8). We therefore trained three machine-learning models that used solely within-platform characteristics to predict the best correlation with any other assay (Fig. 1d). We observed that many protein targets are unlikely to achieve even weak cross-platform concordance (predicted *r* > 0.1; Fig. 1d) when applying the models to the entire set of proteins covered by each technology. This included 13.5% (*n* = 966; Seer XT), 45.5% (*n* = 2,453; Olink HT) and 39.1% (*n* = 4,194; SomaLogic 11k) of protein assays. These findings demonstrate the need to further improve assay performance, for example, sensitivity, and more specific selection of protein targets reliably detectable in blood in the general population.

**Extended Data Fig. 5:**
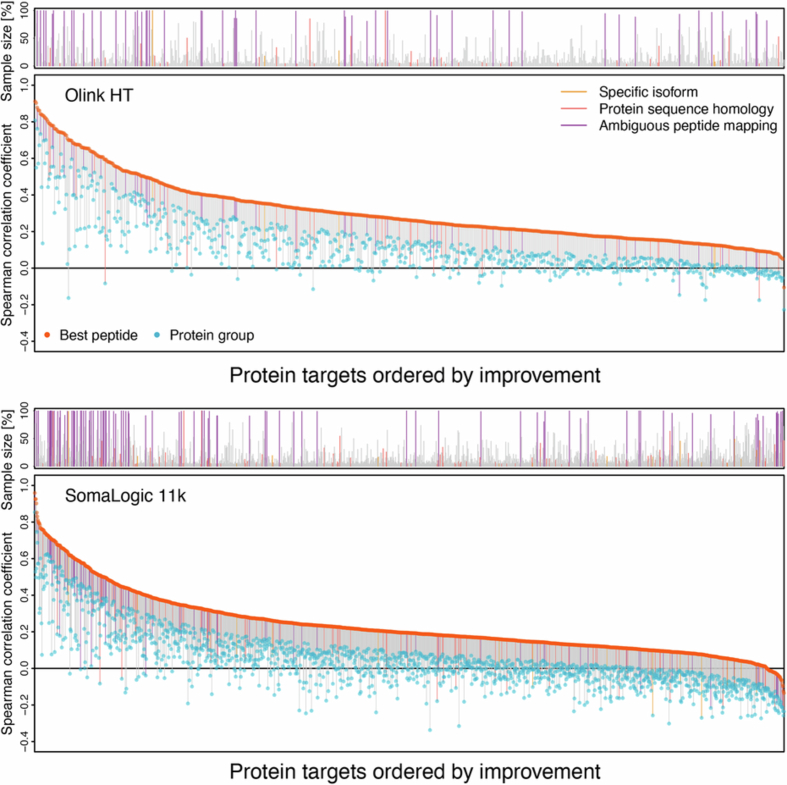
Peptide-level resolved cross-platform correlation of protein targets. Correlation coefficients that improved >0.1 when comparing measurements for overlapping protein targets based on Olink HT (top) or SomaLogic 11k with peptide level (orange dot) instead of protein group measurements (blue dot). Line charts on top of each plot indicate the sample size for the respective best-correlating peptide. Color code refers to possible explanations for the improvement in correlation coefficients (Supplementary Note 1). Orange dots represent the Spearman correlation with the best-correlating MS peptide; blue dots represent the correlation at the protein group level. Line charts indicate the detection rate (fraction of n = 1,396 samples with nonmissing measurements) for the respective best-correlating peptide. Categories (specific isoform, protein sequence homology, ambiguous peptide mapping) are based on bioinformatic annotation of the best-correlating peptide and are mutually exclusive.

**Extended Data Fig. 6:**
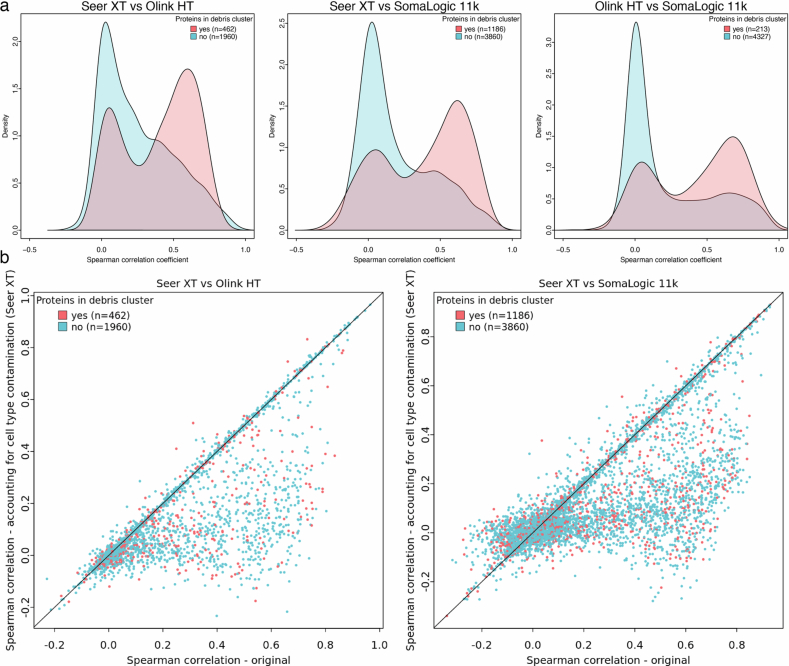
Cross-platform correlation gradients stratified by whether proteins belonged to a cluster likely attributed to cell contamination (‘debris’). **a**, Kernel density estimates of Spearman correlation coefficients for proteins assigned to a debris cluster (pink; Seer XT–Olink HT: n = 462 proteins; Seer XT–SomaLogic 11k: n = 1186, Olink HT-SomaLogic 11k: n=213) versus all other overlapping proteins not assigned to a debris cluster (turquoise; Seer XT–Olink HT: n = 1960; Seer XT–SomaLogic 11k: n = 3860; Olink HT-SomaLogic 11k: n=4327). Debris cluster assignment was based on proteins with r^2^ > 0.8 to known blood cell contamination markers. **b**, Paired comparison of Spearman correlation coefficients before and after correction for blood cell contamination markers in MS-based measurements (n = 1,396 individuals) comparing to the Olink HT platform (left) or the SomaLogic 11k platform (right). Only MS-based values were corrected. Each point represents one protein target; color indicates membership in the blood cell contamination cluster.

Discordance of measurements was further only rarely explained by differential isoform targeting or measurement specificity of affinity reagents (Extended Data Fig. 5 and Supplementary Note 1). We also identified clusters of highly correlated but biologically distinct proteins across platforms (*n* = 28 MS clusters; *n* = 160 SomaLogic 11k; *n* = 14 Olink HT), the largest of which in each case was enriched for markers of blood cell contamination^27^ (odds ratio = 9.13; *P* < 1.1 × 10^−17^; Extended Data Fig. 6 and Supplementary Note 1).

### Rare to common pQTLs

**Fig. 2:**
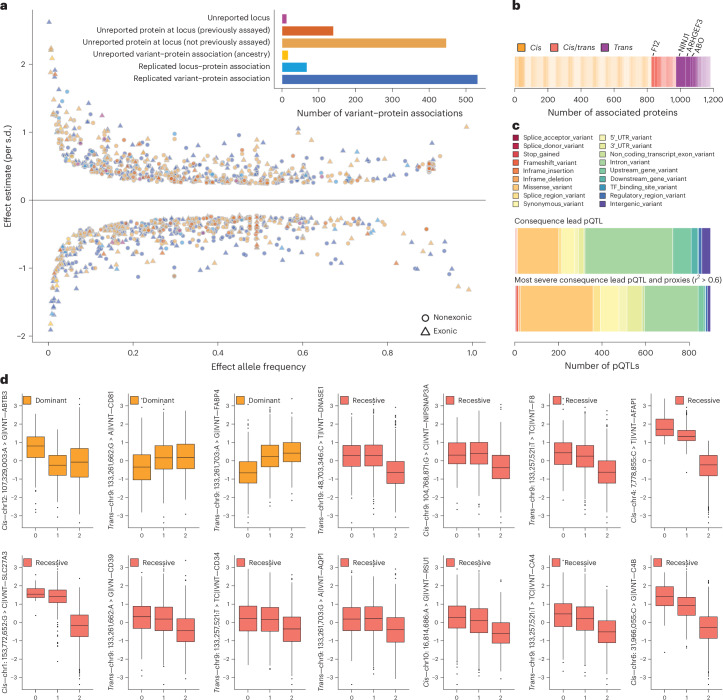
Rare and common pQTLs. **a**, Scatterplot opposing effect allele frequency and effect estimate for associated genetic variants. Symbols (triangles and circles) indicate whether the variant is based on genotype imputation (nonexonic) or WES. The inset quantifies the extent to which associations have been reported previously and is used as a color gradient in the scatterplot. **b**, Bar chart displaying the number of pQTLs inferred from LD-based clumping across protein targets. pQTLs with strong evidence for pleiotropy have been annotated. **c**, Bar charts showing the functional annotation of identified genetic variants or proxies thereof (bottom). **d**, Selected genetic variant–protein associations with evidence for nonadditive genetic effects. Color codes indicate the likely mode of inheritance based on the reference allele. Each box plot is derived from *n* = 1,429 individuals (biological replicates from distinct study participants). Groups 0, 1 and 2 on the *x* axis represent homozygous reference, heterozygous and homozygous alternative allele carriers, respectively. Box plots show median (centerline), IQR (box bounds) and 1.5× IQR (whiskers); points beyond whiskers are individual outliers.

We identified 1,217 independent, significant (*P* < 8.7 × 10^−12^) associations between 1,016 genetic loci and plasma levels of 978 protein groups measured by MS (of 5,768 detected in >70% volunteers) by integrating genome-wide (minor allele frequency (MAF) > 1%) and exome-wide (minor allele count (MAC) > 5) association results using statistical fine-mapping (Fig. 2a, Supplementary Table 9 and Methods). This included 94 variant–protein group pairs associated with >1 s.d. change per effect allele (MAF range = 0.2–41.0%). More than 90% (*n* = 1,026) associations had support from three or more independent peptides, corroborating a strategy that minimizes artificial findings by omitting genetically induced variant peptides from protein group quantification^15,17^ (Methods). Notably, protein groups associated with genetic variants were almost fourfold (odds ratio = 3.89; *P* < 6.8 × 10^−38^) and threefold (odds ratio = 2.83; *P* < 7.9 × 10^−8^) enriched for those predicted to be secreted to blood or the ECM, respectively, supporting the notion that proteins belonging to these compartments are most accessible for blood-based pQTL discovery.

Half of all identified associations (*n* = 617) have not been previously reported (Fig. 2a and Supplementary Table 9), in part explained by the unique coverage of proteins by MS (*n* = 460), but also including 13 loci never linked to plasma protein levels so far, demonstrating the potential of deep MS-based platforms to considerably expand the detection of pQTLs. This was even more apparent considering the 143 associations (*n* = 42 close to the protein-coding gene—‘*cis*’) we identified at previously reported loci and for which we established yet unreported protein associations despite the respective protein target being captured in studies of up to 30 times larger than ours (Fig. 2a). A total of 17 unreported associations were most likely explained by strong differences in allele frequencies across ancestries, being common in our study of volunteers from South Asian ancestry but rare (MAF ≤ 0.5%) in the predominantly white-European ancestry participants of previous studies. This included a common synonymous variant (chromosome (chr)17: 2,042,053:C > A; MAF = 10.0%) associated with plasma levels of esterase OVCA2 (*β* = −0.51; *P* < 2.1 × 10^−16^) that is almost absent in non-Finnish European populations (gnomAD MAF = 0.01%). An additional 43 previously reported variants had effect allele frequencies of >10-fold higher compared to white-European ancestries. For example, the missense variant chr14: 105,737,776:C > T (p.G396R) in *IGHG1* (MAF = 16.3% in South Asians; gnomAD non-Finnish European MAF = 0.06%) is associated with lower plasma levels of bromodomain adjacent to zinc finger domain 1B (BAZ1B) and has been associated with susceptibility to systemic lupus erythematosus in individuals of East Asian ancestry^28^.

We then sought to distinguish specific from pleiotropic regulatory variants (Fig. 2b) by clumping variant-level associations across the proteome into 898 pQTLs. We found that most pQTLs likely acted on the respective protein-coding gene in proximity (±500 kb; *n* = 794 *cis*-pQTL), including 29 *cis*-pQTLs being associated with two and up to three distinct protein groups. For example, a pQTL proxied by chr19: 38,326,336:T > C and chr19: 38,304,610:G > A (*r*^2^ = 0.6) was significantly associated with lower levels of protein phosphatase 1 regulatory subunit 14A (*β* = −0.48; *P* < 1.8 × 10^−24^) and immortalization-upregulated protein (*β* = −0.46; *P* < 9.7 × 10^−26^). While the association with protein phosphatase 1 regulatory subunit 14A has previously been reported^3,4,10,11,13^, the association with immortalization-upregulated protein is unique to MS-based platforms and illustrates the need for additional coverage of the plasma proteome to establish specificity even for *cis*-pQTLs.

The remaining pQTLs (*n* = 104) associated with distally encoded (‘*trans*’) proteins (Fig. 2b and Supplementary Table 9), including 43 pQTLs with *cis* and *trans* associations, possibly suggesting a role of the *cis*-protein in elucidating *trans* effects. For example, a *cis*-pQTL proxied by the upstream gene variant chr5: 177,413,083:G > GC for factor XII (*β* = 0.96; *P* < 4.3 × 10^−132^) was associated with a total of 20 protein groups more than 23-fold enriched for members of the complement and coagulation cascade (fold change = 23.8; *P* < 2.2 × 10^−6^). A finding in line with the central role of factor XII activation in initiating the intrinsic pathway of the coagulation cascade that is responsible for blood clot formation after endothelial injury. In contrast, the 60 protein groups associated with the most pleiotropic *trans*-pQTL (Supplementary Table 9), proxied by a 3′-UTR variant for *NINJ1* (chr9: 93,122,219:A > G), were not enriched for any pathway, leaving the possibility of a platform-specific analytical effect.

Functionally annotating pQTLs or their proxies (*r*^2^ > 0.6) revealed protein-altering variants (PAVs) for 38.8% of *cis*-pQTLs (*n* = 325) and 44.6% of *trans*-pQTLs (*n* = 34; Fig. 2c). About 30% of *cis*-pQTLs mapping to PAVs recorded in ProtVar^29^ were predicted to destabilize (ΔΔG > 2 kcal mol^−1^) the associated protein, exemplified by chr20: 24,971,511:G > A (p.L163F) in adipocyte plasma membrane-associated protein (*β* = −0.99; *P* < 1.6 × 10^−24^; ΔΔG = 10.3). Additional 651 pQTLs mapped to variants likely (posterior inclusion probability > 10%) affecting gene expression in one or more tissues^30^, including 545 *cis*-pQTLs aligning with corresponding *cis*-eQTLs (Supplementary Table 9). Combined with the effects of PAVs, this pointed to the potential underlying mechanisms for 85.8% of all identified pQTLs.

#### Nonadditive genetic effects for *cis*-pQTL and *trans*-pQTL

Among all identified pQTLs, we observed robust support for autosomal dominant (*n* = 3) or recessive (*n* = 11) effects with respect to the reference allele that were also supported by two or more peptides (Fig. 2d and Supplementary Table 10). This included six nonadditive effects close to the protein-coding gene that have not been described to date. For example, homozygous carriers of the common G allele for chr1: 153,772,652:G > C had 1.64 s.d. units lower plasma levels of long-chain fatty acid transport protein 3 (FATP-3 or SLC27A3) compared to carriers of the minor C allele. SLC27A3 activates long-chain fatty acids^31^ and a burden of rare loss-of-function or missense variants has previously been associated with lower asthma risk^32^. Nonadditive effects in *trans* were exclusively explained by recessive effects on proteins involved in glycosylation, namely α1-3-galactosyltransferase (ABO, for example, through chr9: 133,257,521:C > CT, *n* = 7) and fucosyltransferase 2 (FUT2, through chr19: 48,703,346:C > T, *n* = 1), which are known to encode blood group status but possess a wide spectrum of protein targets.

### Cross-platform concordance of pQTLs

**Fig. 3:**
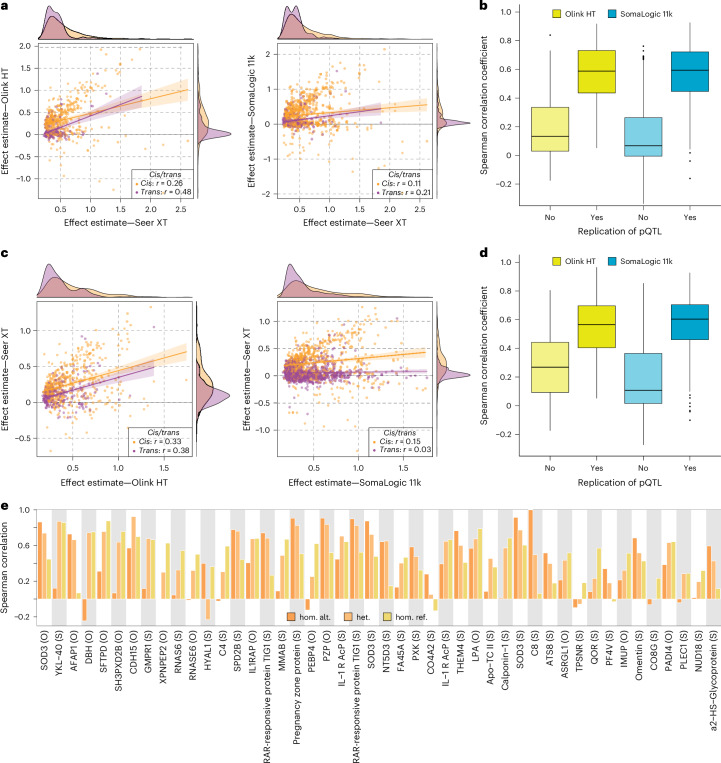
Cross-platform consistency of pQTLs. **a**, Effect estimates for 510 (Olink HT) and 1,009 (SomaLogic 11k) variant–protein associations discovered using MS that overlapped with affinity-based measurements. Correlation coefficients are given stratified by *cis*-pQTLs and *trans*-pQTLs. Shaded bands represent 95% confidence intervals around the linear regression line. Spearman correlation coefficients (*r*) for *cis*-pQTLs and *trans*-pQTLs are shown separately; no adjustment for multiple comparisons was applied within this panel. Association testing was performed using linear mixed models (REGENIE (v4.0)), two sided. **b**, Box plots indicating the distribution of Spearman correlation coefficients of protein targets stratified by whether or not the associated genetic variant replicated using affinity-based techniques. **c**,**d**, Same as **a** and **b**, respectively, but now using 661 (Olink HT) and 2,096 (SomaLogic 11k) genetic variant–protein associations discovered using affinity-based techniques and testing for replication using MS-based measurements. **e**, A total of 46 variant–protein pairs with robust statistical evidence (Supplementary Note 2) that the correlation with MS-based measurements differed according to the genotype. Box plots show median (centerline), IQR (box bounds) and 1.5× IQR (whiskers); points beyond whiskers are individual outliers. Olink HT, *n* = 510 variant–protein associations; SomaLogic 11k, *n* = 1,009 (**b**). Olink HT, *n* = 661; SomaLogic 11k, *n* = 2,096 (**d**). Measurements are from *n* = 1,396 individuals with data on all three platforms. hom., homozygous; alt., alternative; het., heterozygous; ref., reference.

We tested for replication of our MS-based discovery effort using two affinity-based assays in the same volunteers (*n* = 510 and *n* = 1,009, genetic variant–protein group associations using Olink HT and SomaLogic 11k, respectively). About half of the MS-based associations (53.5% for Olink HT and 43.5% for SomaLogic 11k) showed at least moderate evidence of replication (concordant effect sizes and *P* < 10^−5^) with low correlation of effect estimates (Fig. 3a and Supplementary Table 11). Lack of replication coincided with poor correlations of the corresponding protein measurements (Fig. 3b and Supplementary Table 12). Accordingly, most nonreplicating associations (Olink HT = 79.8%; SomaLogic 11k = 83.3%) included protein targets for which we could not identify any genome-wide significant (*P* < 5 × 10^−8^) *cis*-pQTL or *trans*-pQTL using affinity-based measurements. An observation partly explained by low detectability of corresponding affinity measurements, with replication rates for the Olink HT platform improving from 17.3% to 66.8% when comparing proteins with the highest (>50%) to the lowest number of values below an estimated LOD (≤5%; Supplementary Table 13).

Replication of 2,757 affinity-based pQTLs (440 *cis*-pQTL and 221 *trans*-pQTL for Olink HT; 684 *cis*-pQTLs and 1,412 *trans*-pQTLs for SomaLogic 11k; Williamson et al.^33^) using MS results revealed lower replication rates (44.0% Olink HT; 22.9% SomaLogic 11k) despite higher correlations of effect estimates for targets overlapping with the Olink HT platform (Fig. 3c and Supplementary Table 14). We note that almost half (45.1%) of nonreplicating *trans*-pQTLs of the SomaLogic 11k platform were accounted for by five highly pleiotropic *trans*-pQTLs we previously related to specifics of the measurement technique^24^.

Nonreplicating pQTL associations across platforms were best explained by poorly correlating protein measurements, restricting the feasibility of efforts to flag artificial signals^34^ (Fig. 3d, Supplementary Table 12 and Supplementary Note 2). We further identified only few examples with robust evidence for genetically conferred assay differences (Supplementary Note 2, Fig. 3e and Supplementary Table 15).

### Intersection of pQTLs with nonproteomics traits in the GWAS Catalog

**Fig. 4:**
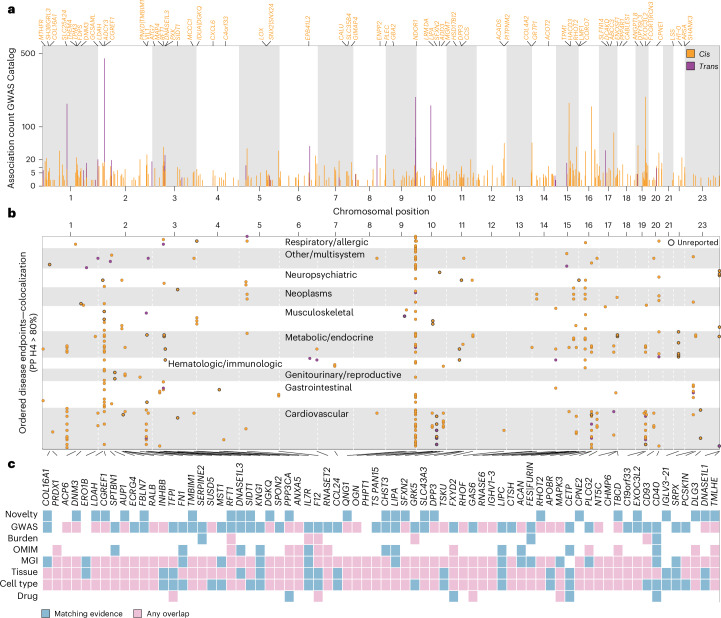
pQTLs link proteins to diseases. **a**, Number of associated nonproteomic phenotypes reported in the GWAS Catalog for pQTLs or their proxies (*r*^2^ > 0.6; displayed by genomic position) discovered using the Seer XT platform. pQTLs mapping to unreported *cis*-pQTLs and associated with ≥5 nonproteomic outcomes were annotated on top. **b**, Results from systematic colocalization analysis of pQTLs with 402 ICD10-coded disease endpoints based on a meta-analysis of FinnGen and UK Biobank^41^ (*y* axis). Only colocalization with strong support of a shared genetic signal between the protein and disease endpoint is shown (PP of H4 > 80% and respective regional sentinels are in high LD (*r*^2^ > 0.8)). Colocalization with so far unreported pQTLs is highlighted by black circles and outcomes have been grouped by anatomical categories. **c**, Additional layers of evidence that may link the colocalizing protein signal with one or more of the associated diseases. The color code indicates whether any entry was reported for the protein-coding gene in each of the databases and blue indicates direct relevance to the disease colocalizing with the *cis*-pQTL. Novelty indicates whether the *cis*-pQTL has been reported; GWAS indicates whether any of the colocalizing disease signals has been reported in the GWAS Catalog; burden indicates matching evidence from a burden of rare predicted loss of function variants^42^; OMIM indicates evidence that a rare disorder linked to the protein encoding gene shares phenotypic similarities with any of the colocalizing diseases^43^; MGI indicates evidence that any mouse phenotype for the protein encoding genes shares phenotypic similarities with the colocalizing diseases based on the MGI initiative^44^; tissue/cell type indicates evidence for enhanced expression of the protein-coding gene in disease-relevant tissues and/or cell types^45^; drug indicates evidence that the protein is the target of an approved drug or a drug in clinical development and the genetic signal is associated with the indication of the drug^48^. MGI, Mouse Genome Informatics.

We then sought to understand whether any of the identified MS-based pQTLs had been associated with downstream consequences transcending the proteome and hence point to putative unknown biological mechanisms. For almost half of the pQTLs (*n* = 425), we observed one or more nonproteomic phenotype reported in the GWAS Catalog^35^ for the same variant or their proxies (*r*^2^ > 0.6; Fig. 4a and Supplementary Table 9). This included 165 unreported *cis*-pQTLs, 68 associated with ≥5 traits, that may guide effector gene assignment (Fig. 4a). For example, we identified a common variant (chr5: 122,073,485:G > C) intronic of *LOX* associated with decreased plasma levels of the enzyme lysyl oxidase (*β* = −0.49; *P* < 1.4 × 10^−38^), the protein product of *LOX*. Lysyl oxidase is secreted by fibrogenic cells and oxidizes lysine residues to promote covalent cross-linking of elastin and collagen fibers to stabilize and insolubilize the ECM^36,37^. A genetically mediated lower activity of LOX, as predicted from lower plasma levels, is in line with separate studies linking the same variant to a thicker cornea^38^, taller height^39^ and less youthful appearance^40^.

### Multilevel evidence for disease-mediating proteins

To robustly identify a potentially disease-mediating role of proteins, we systematically tested whether identified pQTLs in G&H also associated with the risk for one or more of 402 diseases in ~1 million people across the UK Biobank and FinnGen biobanks^41^. We identified a total of 690 pQTL–protein–disease links with strong evidence for a shared genetic signal (posterior probability (PP) > 80%) encompassing 92 unique pQTLs (70 *cis*-pQTLs) and 136 diseases (Fig. 4b and Supplementary Table 16). While the restricted phenotype and disease overlap may have accounted for fewer associated pQTLs as compared to the intersection with the GWAS Catalog, nine pQTLs (seven *cis*-pQTLs) were exclusively but robustly linked to disease risk in the systematic meta-analysis. This illustrates the need to complement disease-centric efforts, as mostly reported in the GWAS Catalog, with comprehensive biobank screens enabled by electronic health record linkage.

**Extended Data Fig. 7:**
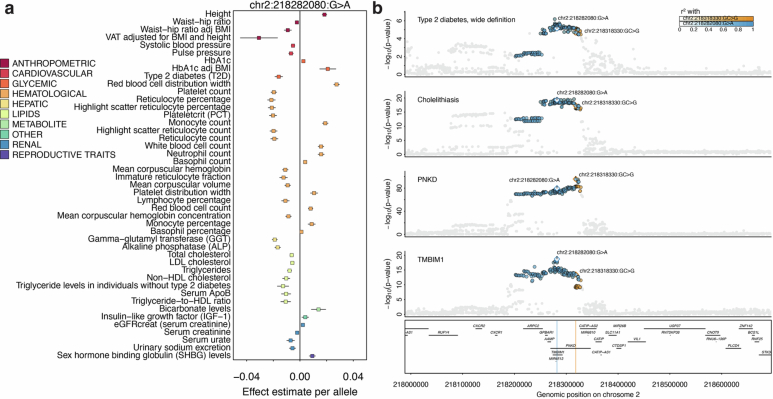
Challenging effector gene annotation at the PNKD/TMBIM1 locus. **a**, Forest plot showing significant (P < 10^−5^) associations between chr2: 218282080:G > A and diverse outcomes. Association statistics are from summary-level GWAS data from the CMD Knowledge Portal (downloaded: 15 April 2025); individual-level data were not used. Shown associations reach P < 10^−5^ are shown. **b**, Stacked regional association plot centered around *TMBIM1* and *PNKD*. The bottom two panels each depict regional association statistics (−log_10_(P)) for plasma levels of transmembrane BAX inhibitor motif-containing 1 (TMBIM1) and PNKD metallo-beta-lactamase domain containing from the present study. Association statistics for cholestithiasis and type 2 diabetes were obtained from a meta-analysis of FinnGen and UK Biobank. Variants were colored based on LD with the respective regional lead variants for each protein. Please note that colors overlap for variants in LD with both (r^2^ > 0.5; greyish). Regional association plots are based on n = 1,413 individuals for plasma protein associations and FinnGen/UK Biobank meta-analysis summary statistics for disease endpoints (n varies by endpoint).

**Extended Data Fig. 8:**
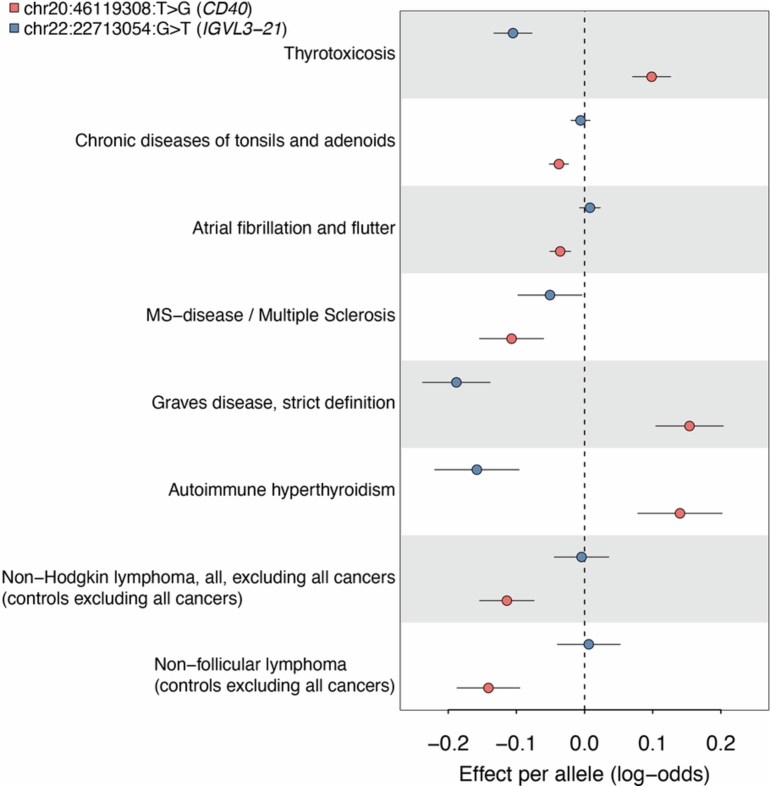
Immune disease risk for variants at the *CD40* and *IGLV3-21* locus. Forest plot showing effect estimates for two *cis*-pQTLs (orange: CD40; blue: IGLV3-21) across diseases with evidence of colocalization for at least one of the two (Supplementary Table 14). Effect estimates were obtained from a meta-analysis of FinnGen and UK Biobank.

A third of the *cis*-pQTLs (*n* = 26) mapped to protein encoding genes not previously implicated through proteogenomic evidence but attributing a causal role to the protein based solely on statistical criteria was insufficient as illustrated for the TMBIM1/PNKD locus and type 2 diabetes risk in Supplementary Note 3 (Extended Data Fig. 7). We therefore collated additional layers of orthogonal evidence, such as rare gene burden analyses^42^, Mendelian disorders^43^, mouse models^44^, as well as tissue and single-cell-type gene expression patterns^45^ (Fig. 4c). Among the most compelling examples were plasma levels of CD40 linked to the risk of diverse immune-related diseases, like multiple sclerosis or autoimmune hyperthyroidism, but also non-Hodgkin lymphoma (Fig. 4b and Supplementary Table 16) through different independent layers of evidence. An observation in line with previous studies linking CD40 not only to various autoimmune but also to other diseases^5,46,47^. CD40 is a target of high pharmacological interest (ten drugs in up to phase II trials across 48 indications^48^), but our genetic results indicated opposing effects across immune-related disorders—agonism for lymphoma and multiple sclerosis but inhibition for autoimmune thyroid disease—and a potential safety signal for CD40 inhibition from an inverse association with atrial fibrillation risk (*β* = −0.04; *P* < 4.1 × 10^−6^; Extended Data Fig. 8).

**Extended Data Fig. 9:**
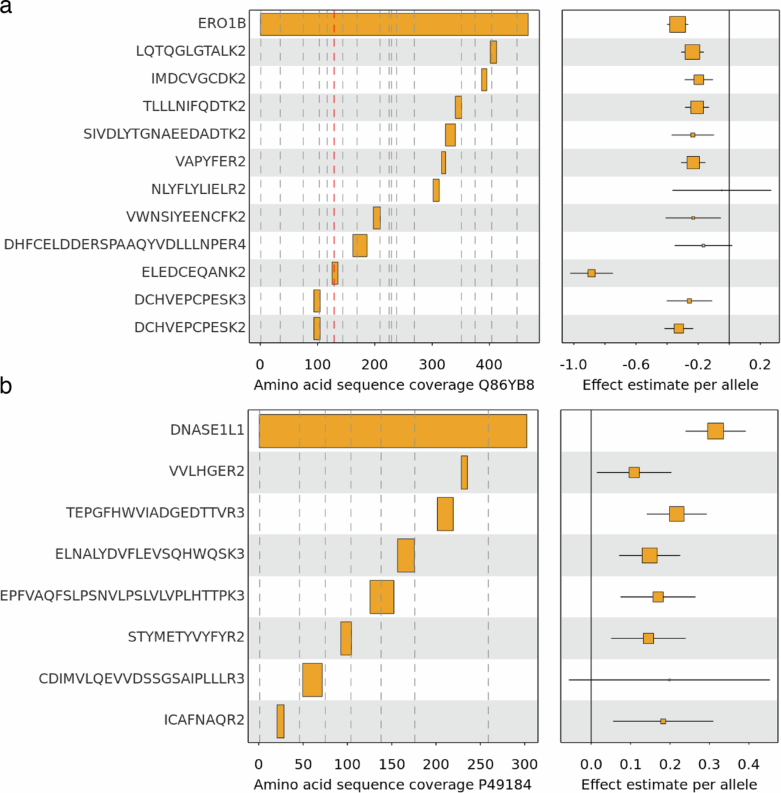
Peptide-level support for the associations of chr1: 236,249,930:T>A with plasma levels of ERO1-like protein beta (ERO1B) and deoxyribonuclease-1-like 1 (DNASE1L1). For each panel, the left side shows the amino acid sequence of each peptide and its location in the protein, with light gray vertical lines indicating exon boundaries of the protein-coding gene; the right side shows effect estimates (rectangles) and 95% confidence intervals (horizontal lines) from linear regression models adjusted for age, sex, ancestry and 20 genetic principal components, in n = 1,413 individuals (biological replicates from distinct study participants). The center of each rectangle represents the point estimate (beta coefficient, in s.d. units), and box size is proportional to the number of observations per peptide. **a**, ERO1B (UniProt Q86YB8). Please note that the peptide mapping to exon 5, ELEDCEQANK2, had not been used for quantification of plasma protein levels, since it encodes a protein-altering variant. **b**, DNASE1L1 (UniProt P49184).

We identified notable examples of unreported or so far poorly understood putative disease mechanisms among the 21 protein–disease(s) connections with evidence from two or more sources (Fig. 4c). Peptide-level evidence thereby helped to clarify underlying genetic mechanisms (Supplementary Note 3). Briefly, we verified exon 5 skipping in *ERO1B*^49^ as a potential mechanism linking lower plasma levels of the gene product to decreased type 2 diabetes risk (PP H4 = 90.8%; *β* = −0.03; *P* < 5.5 × 10^−10^; Extended Data Fig. 9a), while we found no evidence for a role of alternative splicing underlying an association between the *cis*-pQTL for deoxyribonuclease-1-like 1 (Extended Data Fig. 9b) and a reduced risk for type 2 diabetes (PP H4 = 89.8%; *β* = −0.04; *P* < 1.8 × 10^−10^).

### IgG antibodies carrying immunoglobulin λ variable 3-21 (IGLV3-21) may predispose to Graves’ disease

**Fig. 5:**
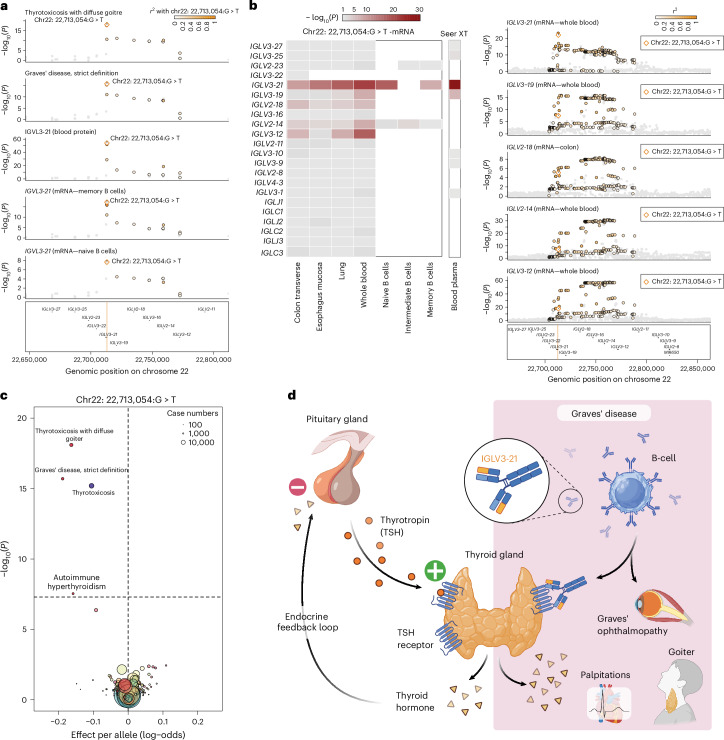
IgG antibodies carrying IGLV3-21 may predispose to Graves’ disease. **a**, Stacked regional association plot centered around *IGLV3-21*. Association statistics for thyroid disorders were obtained from a meta-analysis of UK Biobank and FinnGen^41^. Plasma protein associations from the present study, and mRNA expression from single-cell RNA expression of B cells from the TenK10K consortium^52^. SNPs (dots) are colored by LD with the lead *cis*-pQTL (chr22: 22,713,054:G > T). **b**, Heatmap (left) and stacked regional association plot (right) demonstrating specificity of the association of chr22: 22,713,054:G > T with other encoded immunoglobulin genes at the locus across B-cell populations and tissues based on GTEx (v8)^66^. White fields in the heatmap indicate missing expression of the gene in the respective tissue. **c**, Association statistics for chr22: 22,713,054:G > T across 402 diseases based on the same meta-analysis. **d**, Schematic of the possible mechanism linking IGLV3-21 expression to Graves’ disease. Association statistics in **a** are from linear mixed model-based genome-wide association testing (REGENIE (v4.0)), two sided, in *n* = 1,413 individuals. Disease association statistics (**a** and **c**) are from the FinnGen/UK Biobank meta-analysis (logistic regression-based GWAS, two sided); *n* per disease endpoint ranges from 100 to >100,000 individuals. mRNA association statistics are from published single-cell eQTL analyses^52^. Colocalization PPs of H4 are from coloc with p12 = 5 × 10^−6^. The horizontal dashed line in **c** indicates genome-wide significance; *P* = 5 × 10^−8^. Schematic in **d** created in BioRender; Pietzner, M. https://biorender.com/v49tz7y (2026).

We identified a common missense variant in *IGLV3-21* (chr22: 22,713,054:G > T; p.Asp68Tyr; MAF = 37.1%) that was associated with lower plasma levels of the protein product IGLV3-21 (only assayed by the Seer XT platform; *β* = −0.60; *P* < 7.3 × 10^−55^) and decreased risk of autoimmune hyperthyroid disease, like Graves’ disease (PP = 99.6%; *β* = −0.19; *P* < 1.9 × 10^−16^; Fig. 5a). The protein association was supported by colocalizing gene expression QTLs in multiple tissues most likely driven by B cells and further was remarkably specific (Fig. 5a–c). We observed no evidence that other immunoglobulin genes encoded close by could explain the signal using bulk RNA sequencing and single-cell gene expression data (Fig. 5b). We further observed no strong evidence for other associated diseases, including thyroid or autoimmune diseases, not only in our analysis (minimum *P* = 3.6 × 10^−3^ for chronic lymphoid leukemia; Fig. 5c) but also in large databases^50^ (Supplementary Table 17). The missense variant overlaps with a complementarity-determining region 2 of IGLV3-21 (ref. ^29^), which, along with other hypervariable regions of heavy chains and light chains, determines the binding activity of antibodies to antigens. IGLV3-21 containing antibodies are produced by B cells^51^ and the same genetic variant associated with lower plasma protein levels specifically associated with lower *IGLV3-21* expression in naive and memory B cells^52^ (Fig. 5a). Autoantibodies produced by B cells that activate the thyroid-stimulating hormone receptor, called TSH receptor antibodies (TRAb), are the cause of Graves’ disease and lead to secretion of thyroid hormones to the circulation that is not under control of a negative endocrine feedback loop producing a persistent hyperthyroid state^53^ (Fig. 5d). Specifically, TRAbs have been shown to be of immunoglobulin class G 1 with λ light chains^54,55^. A role of IGLV3-21 in TRAb formation, rather than its plasma levels per se, was further supported by a directionally concordant effect of the *cis*-pQTL variant (chr22: 22,713,054:G > T) on serum titers of TRAb (*β* = −0.08; *P* < 5.2 × 10^−4^) in the FinnGen cohort (*n* = 3681; downloaded on 27 October 2025) but not titers of other autoantibodies linked to autoimmune thyroid disease (Supplementary Table 18).

If validated, our findings may guide disease-modifying treatment strategies. First-line antithyroid medications are associated with ~50% relapse rates at 12–18 months^53^, frequently necessitating radioactive iodine (I-131) or thyroidectomy. Targeting B cells is actively pursued to preserve thyroid function in patients with Graves’ disease informed by the success of the treatment of other autoimmune diseases^56,57^ like multiple sclerosis^58^. We provide human genetic evidence that, in contrast to other proposed B-cell-related targets, like CD40 (Extended Data Fig. 8), specifically targeting B cells that express IGLV3-21 could provide an alternative route that may minimize adverse effects triggering the onset of other autoimmune disorders, as illustrated for CD40. The pQTL’s concordant effect on *IGLV3-21* expression in circulating B cell populations further supports a model in which B cells incorporating IGLV3-21 are selectively depleted from blood. We note that isoforms of IGLV3-21, IGLV3-21*01 and IGLV3-21*02 have so far been only implicated in the development and prognosis of chronic lymphatic leukemia^59^ and vaccine-induced immune thrombotic thrombocytopenia^60^, respectively.

## Discussion

Profiling the abundance of thousands of proteins from blood can guide the identification of disease mechanisms, including potential avenues for developing pharmacological interventions, in living humans when successfully linked with germline variation in the genome. Here we use three complementary plasma proteomic technologies to demonstrate that (1) poor cross-platform agreement is technically driven (predicting ~40% affinity-based measures being unlikely to achieve concordance), (2) pQTL discovery benefits from ancestral and technical diversity, yielding >600 associations unreported in studies >30 times larger, and (3) unambiguously linking proteins to disease requires broad coverage of the proteome and phenome integrated with orthogonal biological evidence.

Combining NP enrichment with MS, we detected >8,000 protein groups in blood at least once, one-third not captured by affinity-based platforms. Technical rather than biological factors thereby contributed to differential platform coverage (for example, amino acid content of proteins and blood cell contamination) and largely accounted for poor cross-platform correlation^13,19,24–26^, particularly assay detectability (Supplementary Note 1). Accounting for measurements below the detection limit improved, although not fully resolved, concordance and pQTL replication, especially for MS-based and antibody-based assays. The latter also exemplified shortcomings of measures of assay precision, such as coefficient of variations (CVs), to judge assay accuracy, that is, providing reliable readouts of the intended protein target, with the SomaLogic 11k platform having the highest precision but poorest cross-platform concordance.

Most pQTL studies have been conducted in participants of European ancestry^7–11^, yet studies in non-European cohorts have demonstrated value even at modest sample sizes^12,16,61^. The relatively small yield of ancestry specific pQTLs in our study compared to cohorts of African individuals^12,62,63^ likely reflects that we were best powered for common variants shared across ancestries^64^. A distinctive feature of our cohort was enrichment of homozygous alternative allele carriers due to a high rate of autozygosity^65^, enabling detection of previously undescribed autosomal recessive and dominant *cis*-pQTL effects on plasma protein levels for proteins with largely uncharacterized roles in human physiology, such as SLC27A3 and NIPSNAP3A.

Integrating pQTLs with disease risk loci can guide drug target development or repurposing, but only if the associated protein can be unambiguously assigned to the disease process. Genetic variants that likely regulate the expression of multiple genes in the proximity represent a particular challenge in these efforts^66,67^, a phenomenon still largely hidden in *cis*-pQTL efforts due to imperfect proteome coverage. We therefore collated multiple additional lines of evidence not only to increase the plausibility of the genetically prioritized link between the protein and disease, including expression in disease-relevant cell types (like the IGLV3-21 example) or concordant evidence from mouse models (like the TMBIM1 example), but also to deprioritize others (like factor XII and peptic ulcers).

Our study is distinguished by the breadth of proteins covered and depth of genetic profiling in an underrepresented population, but multiple limitations need to be considered when interpreting our results. First, while we applied rigorous correction for multiple testing, replication of results in independent studies, including other ancestral groups and nonadditive effects, are warranted. Second, we observed evidence that biophysical properties of proteins/peptides were associated with the coverage of MS-based and affinity-based assays, indicating analytical biases that warrant further investigation^68^. The semiquantitative nature of each of the assays further requires validation using targeted assays. Third, while our approach to genetically link proteins to diseases distinguished by using separate studies and rigorous methods, mismatches in linkage disequilibrium (LD) structure might have led to the underestimation of truly shared signals. Finally, plasma proteomics provides a readout of protein abundance in blood, which might be a reliable readout for disease-relevant mechanisms in cell types or tissues, when combined with human genetic inference, but might miss genetic effects altering protein function but not abundance.

## Methods

This research was conducted in accordance with the Declaration of Helsinki. Ethical approval for the G&H study was granted by the NRES Committee London—South East (reference 14/LO/1240) on 16 September 2014. Queen Mary University of London is the sponsor and data controller. All participants provided written informed consent.

### Study design

G&H is a prospective cohort of more than 70,000 individuals of self-reported Bangladeshi and Pakistani ancestry participants living in the United Kingdom, recruited from age 16 onwards since 2015 (ref. ^21^). Participants completed a brief questionnaire, provided a saliva sample for genotyping and sequencing, and consented to recall and longitudinal linkage to National Health Service (NHS) electronic health records (primary care, secondary care, cancer registry, death registry). Participants received reimbursement for time and travel for recall visits (as per UK NIHR guidance) but no other financial compensation. The current study comprised 1,464 recalled participants. Blood was sampled using ethylenediaminetetraacetic acid tubes to prevent clotting and processed within ≤1 h and stored at −80 °C.

### Proteomic profiling

#### Seer Proteograph

We performed proteomic profiling in 1,612 plasma samples from 1,464 G&H individuals using the Proteograph XT Assay^69,70^. Proteins were quantitatively captured through NP-associated protein coronas, then denatured, reduced, alkylated and enzymatically digested with trypsin and LysC. Resulting peptides were purified, quantified, vacuum dried overnight and reconstituted at 50 ng µl^−1^.

Peptides (400 ng per injection; 8 µl) underwent data-independent acquisition (DIA) analysis using a Vanquish Neo nanoLC coupled to an Orbitrap Astral mass spectrometer (Thermo Fisher Scientific). Peptides were separated using a trap-and-elute configuration (Acclaim PepMap 100 C18 trap column; 50 cm µPAC analytical column) at a flow rate of 1 µl min^−1^ over a 22-min gradient from 5–25% solvent B (0.1% formic acid in acetonitrile), totaling a 33-min run. MS used MS1 scans (380–980 *m*/*z*; Orbitrap detector; resolution = 240,000; 0.6 s cycle; 500,000 ion AGC) and 200 fixed-window MS2 DIA scans (150–2,000 *m*/*z*; 3 Th isolation windows; Astral detector; 25% collision energy; 50,000 ion AGC).

DIA MS data were analyzed using the Proteograph Analysis Suite’s cloud pipelines^69^, using the DIA-NN search engine (v1.8.1)^70^ in library-free, match-between-runs mode. MS/MS spectra were matched against an in silico-generated spectral library based on human protein entries (UniProt UP000005640_9606), incorporating cohort-specific protein variants with MAC ≥5. Search parameters included trypsin digestion (one missed cleavage), N-terminal methionine excision, fixed cysteine carbamidomethylation, peptide length of 7–30 amino acids, *m*/*z* precursor mass range of 300–1,800, *m*/*z* fragment ion range of 200–1,800 and mass accuracy set of 3 ppm (MS1) and 8 ppm (MS2). Precursor and protein group false discovery rate thresholds were set at 1%. Precursor-level data were annotated as ‘plus’ (all variations retained), ‘minus’ (all variable sites purged) or ‘reference’ (only alternate variants purged), following ref. ^17^. Libraries were filtered at MAC ≥ 5 and MAF ≥ 1%. We used the ‘MAC5’ precursor data for all analyses, including for observational correlations with measurements from other platforms. While this led to a drop in precursors supporting protein group assembly, measurements were still highly correlated (*r* = 0.99 across all measurements), indicating a minimal loss in information.

Raw precursor intensities from DIA-NN were normalized using external synthetic peptide standards (PepCal) spiked into samples to account for LC–MS instrument drift. PepCal peptides were quantified through targeted extraction, quality filtered based on retention time, intensity variation (CV) and completeness, and converted into fold-change relative to median peptide intensities across all runs. Normalization factors were calculated using the median fold change across high-quality PepCal peptides, further smoothed over every five consecutive runs. Normalized precursor intensities per (biosample, NP) pair were aggregated into protein-level intensities for each biosample using the MaxLFQ algorithm (fast_MaxLFQ, R iq package^71^), minimizing variance in protein group intensities. Protein-level intensities were batch corrected at the Proteograph plate level using ‘limma’s removeBatchEffect’ function.

After batch correction, 8,079 protein groups were detected in at least one sample, with 5,768 detected in ≥70% of samples. Median CV was 22.8% (IQR = 17.8–30.6%) based on a replicate sample per plate (*n* = 7,089 protein groups). Sixty-seven outlier samples were excluded based on the Mahalanobis distance from the first four principal components (*P* < 3.1 × 10^−5^); for multiply measured samples, the first measurement was retained, leaving 1,429 samples. MS measurements were validated against 17 clinical laboratory markers and body mass index from electronic health records using linear regression (14/18 showed *P* < 0.05/18; Supplementary Table 19). For genome-wide association studies (GWAS), 5,768 protein groups with ≤30% missing values were imputed using miceRanger (v1.4) and rank-inverse normal transformed.

#### Olink Explore HT

Proteomic profiling with the Olink Explore HT platform was performed in 1,641 samples from 1,447 individuals. Olink relies on proximity extension assays in which pairs of antibody-conjugated oligonucleotides bind their target, enabling hybridization, amplification and relative quantification by next-generation sequencing; detailed methodology is described elsewhere^72^. Samples were flagged as failed if <10k read counts were present, or if incubation, extension or amplification controls had <150 counts. Nineteen samples were excluded due to >50% assay failures or >50% assays with counts below the negative control average. Normalized protein expression values were generated by extension-control normalization, log_2_ transformation and plate-control normalization. Median CV across all protein targets was 30.55% (IQR = 16.35–46.53%).

#### SomaLogic 11k (v5)

SomaLogic relies on modified DNA aptamers that recognize target proteins and the detailed methodology is described elsewhere^73^. Scaling factors account for intraplate and interplate effects through hybridization normalization, median signal normalization, plate-scale normalization and adaptive normalization by maximum likelihood. Four outlier samples (median RFU >3 s.d. from the population mean) were excluded. Median CV across all protein targets was 7.71% (IQR = 5.54–12.28%).

### Genotyping and imputation

Genotyping was performed using genomic DNA extracted from saliva samples obtained through Oragene saliva sampling kits. Individuals were genotyped on the Illumina GSA (v3) chip + extra multidisease content. Genotype calling used Illumina GenomeStudio (v2.0) with automated and manual GenTrain clustering on 1,970 high-quality samples, yielding >99% call rates at 637,829 SNPs applied to ~50,000 samples. Of 54,206 genotyped samples, the removal of those with missing NHS numbers, sex discordance or duplicate status yielded 51,176 individuals at 608,329 autosomal SNPs (genotyping rate > 99.9%).

After the removal of rare variants (MAF < 0.0001), palindromic variants and indels, genotypes were imputed to the TOPMed-r3 multi-ancestry imputation panel to genome build hg38 using the TOPMed imputation server^74^. Postimputation quality control (QC) retained biallelic autosomal variants with MAF > 0.01, INFO > 0.7, <10% missingness and no Hardy–Weinberg equilibrium deviation (*P* < 1 × 10^−15^), dosages outside expected ranges (0–0.1, 0.9–1.1, 1.9–2.0) were set to missing, and individuals with >10% missing genotypes were removed. Genetic duplicate samples were identified with KING^75^—ten pairs of probable identical twins were identified, and one of each pair was removed. Using principal component analysis (PCA), we identified and excluded <10 ancestral outliers who did not cluster with South Asian ancestry reference samples from the Human Genome Diversity Project and 1000 Genomes Project^64^. After a clustering-based procedure to estimate categorical ancestry groupings (Bangladeshi or Pakistani), a further 62 participants were excluded due to ambiguous ancestry. We further excluded participants with missing covariate (age and sex) information, and those not included in the proteomic experiment resulting in a final set of 1,413 participants for genome-wide association testing.

### Whole-exome sequencing (WES)

The Broad Institute performed ‘Standard Germline Exome v6’ using Twist exome capture reagents and Illumina 150 bp PE NovaSeq 6000 sequencing. BWA-MEM was used to map to the reference genome hg38 with ALT contigs to produce single-individual gVCF and cram output files. Preprocessing and variant calling were performed using the Exome Germline Single Sample (v3.0.0) pipeline^76^ using Picard (v2.23.8)^77^, GATK (v4.2.2.0) HaplotypeCaller^78^ and SAMtools (v1.11)^79^. The Broad Institute performed basic QC statistics and delivered crams with >85% bases at >20× Twist bait target coverage. The chrY and MT variants were not called, and chrX variants were called diploid for females and males.

Sample QC was applied to remove those with <85% bases at >20× coverage in Gencode exons, with the contamination estimate freemix of >0.03, self-stated sex that did not match biological sex inferred from exome data (and could not be reconciled), without a valid NHS number, and sample duplicates (the lowest coverage sample(s) were removed). Joint genotype calling was performed on these samples using HAIL and GATK GenotypeGVCFs using the Broad Institute Joint Genotyping pipeline^76^. WES crams were compared to 44,396 Illumina GSA (v3) chip genotyping samples by using 3,596 common (MAF > 0.001) SNPs that are in both WES and GSA hard-called genotypes (without imputation) to identify high-confidence matches. WES samples with mismatches were removed after comparing them to the GSA data as likely recruitment or laboratory errors. We further removed samples not predicted to be of South Asian ancestry based on PCA and reference samples from the 1000 Genomes Project. Individuals were stratified into Bangladeshi, Pakistani and other South Asian groups based on PCA. Samples were further excluded if the number of SNVs, transition/transversion ratio, number of transitions, number of transversions, number of insertions, number of deletions, insertion/deletion ratio were outside the median ±6 median absolute deviations (MAD) compared to samples from the same population, or if the heterozygote/homozygote ratio, heterozygosity rate was higher than the median + 6 MADs (to avoid removing samples with high autozygosity).

Variant QC used a random forest classifier trained on chr20 with true positives (1000 Genomes Project, Omni2.5, Mills INDEL, HapMap3) and false positives (QD < 2, FS > 60, MQ < 30) as described^76^. Variants were binned by classifier score; bins of ≥77 for SNVs (true-positive rate = 98.76%) and ≥57 for INDELs (true-positive rate = 95.65%) were retained. Genotype-level QC applied thresholds of DP ≥ 10, GQ ≥ 20 and hetAB ≥ 0.2 for SNVs (random forest bin ≥ 80), and hetAB ≥ 0.3 with call rate of ≥0.95 for INDELs (bin ≥4 0). For subsequent genetic analyses, we retained 1,418 participants with available MS-based proteomic data passing QC.

### Cross-platform protein target mapping and annotation

We extracted from each vendor UniProt identifiers^80^ for protein groups (MS) or affinity targets that resulted in a list of 14,757 unique identifiers (please note that some MS-based protein groups map to multiple UniProt identifiers). We used UniProt identifiers, omitting isoform information that was only available for MS-based assays. For each unique UniProt identifier, we mapped all platform-specific IDs that mentioned the corresponding UniProt identifier in their descriptions to identify unique and overlapping targets across proteomic platforms. We subsequently used different databases^45,80–82^ to annotate protein-coding genes, their genomic position (GRCh build 38), different protein characteristics, tissue-type and cell-type expression patterns of protein-coding genes, as well as predictions of secretome locations. Missing information on the genomic location of protein-coding genes was manually obtained from GeneCards^83^.

We implemented light gradient-boosting machines in a tenfold cross-validation scheme to predict platform coverage for each technology by contrasting with 20,405 proteins encoded in the human genome. For each protein, we computed different measures of amino acid content (count and percentage; percentage of hydrophobic, polar, charged, positively charged, negatively charged, aromatic, aliphatic, small and tiny amino acids) of the canonical protein sequence stored in UniProt^80^. We further obtained estimates of their isoelectric point^84^.

### Statistical analysis

We computed missingness rates for protein targets across platforms based on missing peptide information using MS-based assays, or by computing a pseudolower limit of detection for affinity-based assays by taking the median signal for buffer samples and adding four times the median absolute deviation measured across repeated blank samples. We computed CVs and associated measures based on a biological replicate measured on each plate across proteomic technologies. We further computed standard distribution measures such as mean, s.d. or median, as well as tested for multimodal distributions using a dip test.

As an indicator of measurement concordance based on semiquantitative assay readouts, we computed Spearman correlation coefficients for all pairwise overlapping targets among 1,396 unique participant samples who had measurements available on all three platforms. We did not impute any values for these analyses. For affinity-based targets, we additionally tested whether stratifying computation of Spearman correlation coefficients by genotypes of associated *cis*-pQTLs improved concordance with MS-based measurements by (1) testing for an interaction effect of the SNP with MS-based measurements and (2) computing *P* values for differences in correlation coefficients as implemented in the R package cocor (v.1.1.4)^85^.

We further computed all within-platform correlations among covered proteins to identify clusters of highly correlated (*r*^2^ > 0.8) proteins. We tested those clusters for the enrichment of previously reported markers of blood cell contamination^27^ using Fisher’s exact test.

For each of the three pairwise-platform comparisons, Boruta feature selection^86^ (v9.0) identified characteristics significantly associated with correlation gradients across overlapping protein targets, incorporating platform-specific QC measures (Supplementary Table 8), genetic findings and protein characteristics. Random forest models were trained to predict Spearman correlation coefficients using tenfold cross-validation (caret and random forest R packages (v7.0 and v4.7)). Final per-platform models used only within-platform QC measures and generic protein characteristics as features, and were applied to predict concordance even for proteins unique to each platform. Analogous frameworks were used to predict pQTL replication, with variant and gene characteristics from VEP (v111) added as features for *cis*-pQTL models.

### Genome-wide association analysis

We ran genome-wide association testing for plasma levels of each of 5,768 protein groups using the REGENIE software^87^ (v.4.0). For step 1, we used an LD-pruned (*r*^2^ < 0.2 or <0.9, respectively) set of common (MAF > 1%) genotyped (GWAS) or sequenced (WES) markers with high coverage (missingness < 1%). We included sex, age, inferred ancestry (Bangladeshi or Pakistani ancestry) and the first 20 genetic principal components as covariates. In step 2, we ran association testing again separately for imputed and sequenced genetic variants using the same set of covariates and default parameters for REGENIE.

### Integrated statistical fine-mapping and novelty assignment

To integrate results from genome-wide (‘GWAS’) and exome-wide association testing (‘ExWAS’), we first extracted regional lead signals (*P* < 8.6 × 10^−12^ correcting genome-wide significance by 5,768 tested protein groups) in a 3 Mb window from each file set separately. After collating associated regions across GWAS and ExWAS results files, we used statistical fine-mapping^88^ as implemented in the R package susieR (v.0.14.2) to identify independent credible sets (maximum *r*^2^ < 0.25). For each region, we retained exonic variants based on exome sequencing or imputed genetic variants otherwise. We sequentially tested for the presence of one to at most ten credible sets, and afterward computed for each iteration the LD among all possible lead credible set variants. We retained the maximum number of credible sets that still delivered approximately independent credible sets. We used combined genetic information on 690 unrelated participants of the G&H cohort with available proteomic data to compute the LD backbone for fine-mapping. We finally filtered the fine-mapping results to retain only credible sets for which the lead variant passed a corrected statistical significance test. This integrated approach allowed us to identify independent coding and noncoding pQTLs in the same region without the additional computational burden of running conditional analysis.

For each identified lead credible set variant–protein group pairing, we intersected the associated region with 17 previous large-scale proteomic studies^4–8,11–15,24,34,89–93^. Associations at unreported regions were classified as ‘new locus’ and those at reported loci but for a different protein target (stratified by whether the protein was previously assayed) as ‘new protein at locus’. For existing locus–protein associations, LD with reported signals (*r*^2^ > 0.1) was used to determine replication. Ancestry-specific signals were flagged by comparing G&H allele frequencies to gnomAD non-Finnish European frequencies.

### Testing for nonadditive effects

For each lead credible set variant–protein pair, we tested for a potential departure from an additive genetic model by rerunning association testing for this pair but introducing another term encoding heterozygous carriers into the model following previous work^94^. Briefly, the additional term is a generic term to test for a significant departure from linearity across the three genotypes and we subsequently assigned potential models of inheritance based on computing log-likelihood ratio tests recoding the genotype according to three different models of inheritance based on the annotated reference allele. To distinguish between truly recessive/dominant effects and other deviations from normality, we additionally computed the gain in significance of either the dominant or additive model as suggested previously and kept only variant–protein association examples with a gain of >1 (ref. ^95^). These analyses were implemented in R (v.4.3.1) using the REGENIE step 1 files to account for relatedness.

### Effector gene assignment

For each lead credible set variant and proxies in strong LD, we queried multiple databases to facilitate effector gene assignment. Those included data from the ABC CATlas^96^, fine-mapped eQTL catalog data^97^, evidence from Hi–C experiments^98,99^, OMIM^43^ or OpenTargets^48^.

To rank candidate effector genes within a locus of interest, we designed a pragmatic but transparent scoring system. We assigned points of 1 if a gene in the locus was linked to the variant if (1) it was the closest gene, (2) it overlaped an exon, (3) the protein target encoded gene (‘*cis*’), (4) it encoded a protein complex or ligand receptor partner (‘*trans*’), (5) there was eQTL evidence (posterior inclusion probability > 0.1), (6) there was evidence from ABC score, and (7) evidence that the section of the genome was implicated by Hi–C to the expression of the gene in a tissue or cell type.

### Phenotypic follow-up of pQTLs

To assess whether pQTLs, that is, genetic signals merged across all protein targets, might have been already reported for nonproteomic traits, we computed LD proxies (*r*^2^ > 0.6) for each lead credible set variant and intersected those with the GWAS Catalog (downloaded on 3 February 2025). For each unique trait, we only kept the genetic variant in highest LD or the pQTL itself to avoid double counting. Before doing so, we filtered the GWAS Catalog results for signals meeting at least genome-wide significance, reporting of risk alleles and omitting blood proteomic studies.

We systematically tested for shared genetic architecture between pQTLs and 402 diseases from a FinnGen/UK Biobank meta-analysis^41^, restricting to endpoints with ≥3 genome-wide signals. Regions with *P* < 10^−6^ were carried forward to colocalization using default priors apart from p_12_ = 5 × 10^−6^ as recommended^100^ under the one-causal-variant assumption. Regional lead variants in low LD (*r*^2^ < 0.8) were treated as distinct signals. This conservative approach may have missed some true-positive colocalizations due to nonmatching LD backbones.

### Reporting summary

Further information on research design is available in the Nature Portfolio Reporting Summary linked to this article.

## Online content

Any methods, additional references, Nature Portfolio reporting summaries, source data, extended data, supplementary information, acknowledgements, peer review information; details of author contributions and competing interests; and statements of data and code availability are available at 10.1038/s41588-026-02697-6.

## Supplementary information

Supplementary Information Supplementary Notes 1–3.

Reporting Summary

Peer Review file

Supplementary Tables Supplementary Tables 1–19.

## Data Availability

The individual-level plasma proteomic, genomic and linked phenotype data are Special Category Data under UK-GDPR (Article 9) and cannot be deposited openly; they are available under controlled access to researchers worldwide via the G&H Use and Access Committee at https://www.genesandhealth.org/researchers/apply-for-access/. Genome-wide summary statistics can be obtained from https://omicscience.org/apps/pGWAS_GandH_seer/.

## Code availability

The analysis code supporting the findings of this study is available on GitHub at https://github.com/comp-med/seer-pqtl-elgh and has been archived on Zenodo at 10.5281/zenodo.20052127 (ref. ^101^).
