# Supplementary material for: Nanoparticle-enriched mass spectrometry proteomics in British South Asians identifies links between genetic variants, plasma protein levels and disease risk
Source: Nat Genet. 2026 Jul 24;58(8):1866–76. doi: 10.1038/s41588-026-02697-6 (PMC13447103; doi:10.1038/s41588-026-02697-6)
Supplement: Supplementary file 1 [file 41588_2026_2697_MOESM1_ESM.pdf]

nature genetics

Supplementary information https://doi.org/10.1038/s41588-026-02697-6

Nanoparticle-enriched mass spectrometry proteomics in British South Asians identifies links between genetic variants, plasma protein levels and disease risk

In the format provided by the authors and unedited

Supplementary Notes

Supplementary Note 1 - *Cross-platform plasma proteomics: technical determinants of coverage and concordance*

The differential coverage of the plasma proteome was to some extent explained by physical properties of proteins and their associated peptides covered by each platform (**Extended Data Fig. 2**). Briefly, machine learning models were moderately able to discriminate proteins captured by each platform out of all reviewed human protein sequences (n=20,405; median balanced accuracy: Seer XT=0.68; Olink HT=0.64; SomaLogic 11k=0.70) based on amino acid composition and derived properties. Proteins captured by affinity reagents were enriched for those with a high percentage of polar amino acids that frequently sit on the surface of proteins and compose epitope binding regions, but profiles differed when contrasting to discriminative properties of 4580 proteins curated by the Human Plasma Protein Project (HPPP)[1]. The latter showed strong resemblance to the importance profile of protein properties to discriminate the coverage of the Seer XT platform (**Extended Data Fig. 2**), including an enrichment of proteins high in aspartate but low in cysteine and serine. We note, that coherent discriminative profiles between the HPPP list and the Seer XT platform might be, at least partly, explained by both using mass spectrometry as an read out and associated analytical biases during chromatographic separation, protein modification and digestion, or ionization of peptides that cannot easily be overcome.

Discordance of protein measurements across platforms has been further suggested to be due to targeting of different isoforms of the same protein[2–6]. To test this hypothesis, we repeated correlation analysis using peptide level data from MS measurements. We observed higher (delta r>0.1) correlation coefficients of plasma levels for 721 and 1401 proteins captured by Olink or SomaLogic, respectively, possibly indicating evidence for preferential targeting of specific epitopes by affinity reagents (**Fig. 1e** and **Extended Data Fig. 5**and **Tab. 5-7**). However, most of these improvements (83.1% and 88.8%) were only seen with peptides detected in less than half of the population (**Fig. 1e**) although the affinity measurements were almost complete, making it hard to distinguish from random improvements. We observed only few examples for protein isoform-specific targeting (best correlating peptide is specific to an isoform: n=5 antibody reagents; n=17 aptamer reagents), unspecific targeting of affinity reagents (best correlated peptide is shared across affinity reagents: n=44 antibody targets:

n=97 aptamer targets), or ambiguities in bioinformatic peptide assignments to protein groups (n=71 antibody targets; n=125 aptamer reagents) explaining poor correlations across protein platforms.

Finally, we observed that cell type contamination during sample preparation may differentially affect plasma proteomic measurements across technologies without modulating cross-platform concordance. Briefly, we identified clusters of highly correlated (r^2>0.8) but biologically distinct proteins for MS-based measurements (n=1544 proteins across 28 clusters), on the SomaLogic 11k platform (n=1139 proteins across 160 clusters), and to lesser extent on the Olink HT platform (n=100 proteins across 14 clusters). The largest cluster from each platform (MS: n=1455; SomaLogic: n=448; Olink: n=60) showed strong overlap of protein targets across platforms and was significantly enriched (odds ratio: 9.13; p-value = 1.1×10^{-17}) for markers of blood cell contamination[7]. We note that proteins within these clusters showed on average higher cross platform concordance compared to the overall set of proteins and correcting for such preanalytical bias reduced cross-platform concordance (**Extended Data Fig. 6**).

The role of technical factors contributing to poor cross-platform correlation have been described in detail elsewhere[2–4], but we add some notable observations. Firstly, accounting for measurements below the detection limit strongly improved assay concordance and pQTL replication but this effect was platform dependent with MS- and antibody-based assays showing strongest improvements. Improving assay sensitivity, including quantification of absolute protein quantities, should therefore be high priority. Secondly, CVs were important factors explaining poor correlations across technologies but must be interpreted in relative terms within a technology. That is, a lower CV for a protein in one technology does not necessarily translate into more reliable measurements. Thirdly, investigating measurement correlations at the peptide instead of protein group-level showed little evidence for affinity reagents preferring certain protein isoforms that may explain poor correlations and further exemplified the need to improve peptide to protein mappings in MS-based efforts. Lastly, differential susceptibility to blood cell contamination during sample preparation may translate to a different impact on downstream phenotype analysis. For example, proteins affected by such effects were depleted for pQTLs in our MS-based effort.

Supplementary Note 2: Assessment of MS-based proteomics for flagging platform-specific pQTL artefacts

Non-replicating pQTL associations were best explained by poorly correlating protein measurements across platforms even when considering dozens of variant and protein characteristics in machine learning-based feature selection (**Fig. 3d and Supplementary Tab. 12**). This suggests that technical factors, such as low detectability, account for most non-replicating pQTLs irrespective of the platform used and questions the feasibility of MS-based scores to flag artificial affinity-based pQTL efforts as proposed recently[8]. For example, among the 81 pQTLs from the two so far largest pQTL efforts proposed to be invalid and captured in our data, we observed only 25 protein targets to correlate sufficiently well (r>0.5) between MS- and affinity-based measurements to allow for such inference (i.e., that both platforms measure the same protein). In general, the extent to which PAVs or alike possibly introduce artificial pQTLs when relying on affinity reagents seemed to be moderate. For example, we identified robust evidence for only 46 out of 1133 instances that correlation coefficients were statistically different according to genotypes of associated cis-pQTLs indicating genetically induced poor assay performance (false discovery rate<5% and significantly different correlation coefficients $p<4.4x10^{-5}$; **Fig. 3e** and **Supplementary Tab. 15**). For less than half (n=20) of the examples, we were able to identify PAVs to possibly explain such effects, i.e., that the altered protein sequence may interfere with the recognition by the affinity reagent. Another explanation might be that those cis-pQTLs tag splicing QTLs producing different protein isoforms.

Supplementary Note 3: Proteogenomic evidence for disease mechanisms

We identified a shared genetic signal between the cis-pQTL chr2:218282080:G>A (p.Pro21Leu; CADD score: 24.2) decreasing plasma levels of transmembrane BAX inhibitor motif-containing 1 (TMBIM1, beta = -0.34; p-value<$1.9x10^{-19}$) and a decreased risk for type 2 diabetes (PP=83.2%; beta = -0,03; p-value<$7.0x10^{-6}$) but an increased risk for cholelithiasis (PP=91.9%; beta = 0.05; p-value<$5.6x10^{-20}$) (**Extended Data Fig. 7**). Our observations align with previous associations at this locus that have been reported for a variety of phenotypes and diseases (n=26) as diverse as blood cell measures, body fat distribution, bone mineral density, colorectal cancer, inflammatory bowel disease, or blood pressure (**Extended Data Fig. 7**).

Confident effector gene assignment, however, has been difficult since multiple functional variants for different genes are in high LD, including completely overlapping candidate genes *TMBIM1* and *PNKD* for both of which we observe cis-pQTLs in high LD (r^2=0.76) (**Extended Data Fig. 7**). The higher posterior probability of a shared signal with the cis-pQTL for TMBIM1 (PP=83.2%) compared to PNKD (PP=50.6%) for type 2 diabetes, even more so for cholelithiasis (91.9% vs 18.4%), aligns with recent evidence that low TMBIM1 by gene knock out can facilitate adipocyte expansion in white adipose tissue rather than adipogenesis, improving obesity-related diseases in mouse models[9]. The latter observation aligns with a decreasing effect of the cis-pQTL on waist-to-hip ratio adjusted for body mass index, a measure of central rather than peripheral fat storage associated with an adverse cardiometabolic health profile[10].

The common exonic variant chr1:236249930:T>A (AF=50.1%) was associated with lower plasma levels of ERO1-like protein beta (ERO1B; beta=-0.32; p-value<6.4×10^{-20}) and a decreased risk for type 2 diabetes (PP H4 = 90.8%; beta=-0.03; p-value<5.5×10^{-10}). The same variant has recently been demonstrated to likely mediate risk for type 2 diabetes by introducing skipping of exon 5[11], which we corroborated using peptide level data for ERO1B (**Extended Data Fig. 9a**). Our findings of a protective effect thereby contrast experimental work suggesting loss of *ERO1B* function leading to reduced insulin biogenesis and secretion in cellular and animal models, and hence a diabetic state[1,2], but align with observations in a diabetic mice model in which overexpression of *Ero1b* in pancreatic beta cells promoted endoplasmic reticulum stress and decreased insulin content of the cells[12]. The functional role of the truncated protein therefore warrants further exploration. Resolving association with peptide level evidence further supported an association between higher (plasma) levels of deoxyribonuclease-1-like 1 (DNASE1L1 or DnaseX; beta=0.31; p-value<4.5×10^{-15}; **Extended Data Fig. 9b**) and a reduced risk for type 2 diabetes (PP H4=89.8%; beta=-0.04; p-value<1.8×10^{-10}) but increased risk for atrial fibrillation (PP H4=88.3%; beta=0.04; p-value<8.6×10^{-6}). An observation in contrast to suggestive evidence that alternate splicing at the locus may explain its link to complex phenotypes[13]. Briefly, DNASE1L1 is a membrane bound DNAase highly expressed in myocytes, including skeletal muscle, a major site of glucose metabolism, and heart[14]. Accumulation of cell free DNA because of cell death can trigger sterile inflammation if not effectively cleared by DNases, a process previously suggested to promote adipose tissue insulin resistance[1]. We note, that we cannot rule out the possibility that the opposing

association observed for type 2 diabetes and atrial fibrillation might be due to different causal genes at the locus, since the cis-pQTL for DNAS1L1 (chr23:154355526:T>C) has also been reported as an cis-eQTL for *RPL10* in the heart[15] or memory T-cells[16].

REFERENCES

1. Geyer, P. E. *et al.* The Circulating Proteome–Technological Developments, Current Challenges, and Future Trends. *J. Proteome Res.* **23**, 5279–5295 (2024).
2. Eldjarn, G. H. *et al.* Large-scale plasma proteomics comparisons through genetics and disease associations. *Nature* **622**, 348–358 (2023).
3. Pietzner, M. *et al.* Synergistic insights into human health from aptamer- and antibody-based proteomic profiling. *Nat. Commun.* **12**, 6822 (2021).
4. Katz, D. H. *et al.* Proteomic profiling platforms head to head: Leveraging genetics and clinical traits to compare aptamer- and antibody-based methods. *Sci. Adv.* **8**, 5164 (2022).
5. Beimers, W. F., Overmyer, K. A., Sinitcyn, P., Lancaster, N. M. & Coon, J. J. A Technical Evaluation of Plasma Proteomics Technologies. 2025.01.08.632035 Preprint at https://doi.org/10.1101/2025.01.08.632035 (2025).
6. Kirsher, D. Y. *et al.* The Current Landscape of Plasma Proteomics: Technical Advances, Biological Insights, and Biomarker Discovery. 2025.02.14.638375 Preprint at https://doi.org/10.1101/2025.02.14.638375 (2025).
7. Korff, K. *et al.* Pre-analytical drivers of bias in bead-enriched plasma proteomics. *EMBO Mol. Med.* 1–23 (2025) doi:10.1038/s44321-025-00309-0.
8. Suhre, K. *et al.* A genome-wide association study of mass spectrometry proteomics using the seer proteograph platform. 2024.05.27.596028 Preprint at https://doi.org/10.1101/2024.05.27.596028 (2024).

9. Zhao, G.-N. *et al.* TMBIM1 is an inhibitor of adipogenesis and its depletion promotes adipocyte hyperplasia and improves obesity-related metabolic disease. *Cell Metab.* **33**, 1640-1654.e8 (2021).

10. Lotta, L. A. *et al.* Association of Genetic Variants Related to Gluteofemoral vs Abdominal Fat Distribution with Type 2 Diabetes, Coronary Disease, and Cardiovascular Risk Factors. *JAMA - J. Am. Med. Assoc.* **320**, 2553–2563 (2018).

11. Atla, G. *et al.* Genetic regulation of RNA splicing in human pancreatic islets. *Genome Biol.* **23**, 196 (2022).

12. Awazawa, M. *et al.* Deregulation of pancreas-specific oxidoreductin ERO1β in the pathogenesis of diabetes mellitus. *Mol. Cell. Biol.* **34**, 1290–1299 (2014).

13. Brumpton, B. M. & Ferreira, M. A. R. Multivariate eQTL mapping uncovers functional variation on the X-chromosome associated with complex disease traits. *Hum. Genet.* **135**, 827–839 (2016).

14. Keyel, P. A. Dnases in health and disease. *Dev. Biol.* **429**, 1–11 (2017).

15. GTEx Consortium. The GTEx Consortium atlas of genetic regulatory effects across human tissues. *Science* **369**, 1318–1330 (2020).

16. Nathan, A. *et al.* Modeling memory T cell states at single-cell resolution identifies in vivo state-dependence of eQTLs influencing disease. https://doi.org/10.1101/2021.07.29.454316 doi:10.1101/2021.07.29.454316.
