# Supplementary material for: Nanoparticle-enriched mass spectrometry proteomics in British South Asians identifies links between genetic variants, plasma protein levels and disease risk
Source: Nat Genet. 2026 Jul 24;58(8):1866–76. doi: 10.1038/s41588-026-02697-6 (PMC13447103; doi:10.1038/s41588-026-02697-6)
Supplement: Supplementary file 2 [file 41588_2026_2697_MOESM2_ESM.pdf]

nature portfolio

Corresponding author(s): Maik Pietzner, David A van Heel, Claudia Langenberg

Last updated by author(s): Jun 17, 2026

Reporting Summary

Nature Portfolio wishes to improve the reproducibility of the work that we publish. This form provides structure for consistency and transparency in reporting. For further information on Nature Portfolio policies, see our Editorial Policies and the Editorial Policy Checklist.

Statistics

For all statistical analyses, confirm that the following items are present in the figure legend, table legend, main text, or Methods section.

n/a	Confirmed	
☐	☒	The exact sample size (n) for each experimental group/condition, given as a discrete number and unit of measurement
☐	☒	A statement on whether measurements were taken from distinct samples or whether the same sample was measured repeatedly
☐	☒	The statistical test(s) used AND whether they are one- or two-sided<br>*Only common tests should be described solely by name; describe more complex techniques in the Methods section.*
☐	☒	A description of all covariates tested
☐	☒	A description of any assumptions or corrections, such as tests of normality and adjustment for multiple comparisons
☐	☒	A full description of the statistical parameters including central tendency (e.g. means) or other basic estimates (e.g. regression coefficient) AND variation (e.g. standard deviation) or associated estimates of uncertainty (e.g. confidence intervals)
☐	☒	For null hypothesis testing, the test statistic (e.g. F, t, r) with confidence intervals, effect sizes, degrees of freedom and P value noted<br>*Give P values as exact values whenever suitable.*
☐	☒	For Bayesian analysis, information on the choice of priors and Markov chain Monte Carlo settings
☒	☐	For hierarchical and complex designs, identification of the appropriate level for tests and full reporting of outcomes
☐	☒	Estimates of effect sizes (e.g. Cohen's d, Pearson's r), indicating how they were calculated

Our web collection on statistics for biologists contains articles on many of the points above.

Software and code

Policy information about availability of computer code

Data collection	No software was used for data collection.
Data analysis	Data analyses were performed using Illumina GenomeStudio version 2.0; TOPMED imputaIon server; KING; Exome Germline Single Sample 3.0.0 pipeline; Picard 2.23; GATK 4.2.2.0;HaplotypeCaller; Samtools 1.1182; REGENIE (v.4.0); DIA-NN search engine (v1.8.1); VEP v111; Further downstream analyses were implemented in R, using packages, iq, limma, miceRanger (v.1.4), cocor (v.1.1.4), Boruta (v.9.0), caret and RandomForest (V7.0 and V4.7), susieR (v.0.14.2),<br><br>The analysis code supporting the findings of this study is available on GitHub at https://github.com/comp-med/seer-pqtl-elgh and has been archived on Zenodo at https://doi.org/10.5281/zenodo.20052127.

For manuscripts utilizing custom algorithms or software that are central to the research but not yet described in published literature, software must be made available to editors and reviewers. We strongly encourage code deposition in a community repository (e.g. GitHub). See the Nature Portfolio guidelines for submitting code & software for further information.

Data

Policy information about availability of data

All manuscripts must include a data availability statement. This statement should provide the following information, where applicable:

- Accession codes, unique identifiers, or web links for publicly available datasets
- A description of any restrictions on data availability
- For clinical datasets or third party data, please ensure that the statement adheres to our policy

The individual-level plasma proteomic, genomic and linked phenotype data are Special Category Data under UK-GDPR (Article 9) and cannot be deposited openly; they are available under controlled access to researchers worldwide via the Genes & Health Use and Access Committee at https://www.genesandhealth.org/researchers/apply-for-access/. Genome-wide summary statistics can be obtained from https://omicscience.org/apps/pGWAS_GandH_seer/.

Research involving human participants, their data, or biological material

Policy information about studies with human participants or human data. See also policy information about sex, gender (identity/presentation), and sexual orientation and race, ethnicity and racism.

Reporting on sex and gender	We defined 'female' and 'male' sex including participants where the recorded sex and sex chromosomes aligned (XX for females and XY for males). The recorded sex was self-reported, and it was not possible to distinguish sex from gender. We acknowledge the importance of distinguishing between sex and gender in research and that chromosomal make-up does not always align with self-identified gender.
Reporting on race, ethnicity, or other socially relevant groupings	We projected principal components from the 1000Genomes project onto Genes & Health genetic data and assigned all participants clustering with individuals of South Asians as Pakistani/Bangladeshi, dropping a total of 62 participants due to ambiguous ancestral assignments. We further established by Pakistani/Bangladeshi ancestry by clustering remaining samples and cross-referencing with self-reported ancestry.
Population characteristics	The baseline assessment was performed at recruitment, upon which participants filled in a brief questionnaire, provided a saliva sample for genotyping and sequencing, and consented to being recalled and to longitudinal linkage to electronic health records (EHR) including data from the UK National Health Service (NHS) primary, secondary care, cancer and death registry. Details of the cohort have been previously described.<br>The current study comprises a subset of 1464 participants from the G&H cohort that were recalled for additional phenotyping and blood sampling (N=1464, mean age 43.4 years (12.2 s.d.), 55.9% females) .
Recruitment	G&H is a cohort of over 70,000 South Asian (Bangladeshi and Pakistani) individuals living in the United Kingdom23 (UK). Inclusion criteria included individuals aged 16 and over, from self-reported Bangladeshi and Pakistani backgrounds. Recruitment has been ongoing since 2015.
Ethics oversight	G&H was approved by the London Southeast NRES Committee of the Health Research Authority (14/LO/1240).

Note that full information on the approval of the study protocol must also be provided in the manuscript.

Field-specific reporting

Please select the one below that is the best fit for your research. If you are not sure, read the appropriate sections before making your selection.

☒ Life sciences ☐ Behavioural & social sciences ☐ Ecological, evolutionary & environmental sciences

For a reference copy of the document with all sections, see nature.com/documents/nr-reporting-summary-flat.pdf

Life sciences study design

All studies must disclose on these points even when the disclosure is negative.

Sample size	The current study comprises a subset of 1464 participants from the G&H cohort that were recalled for additional phenotyping and blood sampling (N=1464, mean age 43.4 years (12.2 s.d.), 55.9% females) . No prior sample size calculations were performed, and sample size was constrained by recall success. Numerous previous pGWAS already indicated sufficient power for pQTL discovery at similar sample size.
Data exclusions	Exclusions were done based on different data entities and may partly overlap: n=62 genetic ancestry; n=67 Seer proteomics (multivariate outlier); n= 19 Olink proteomics (>50% missingness); n=4 SomaScan proteomics (outlier RFU values)
Replication	We partially replicated genetic results by cross-referencing across three proteomics technologies and referencing to >30 previous pQTL studies.
Randomization	N/A - randomization occurred naturally as genetic variants were the exposure.
Blinding	N/A - genetic association testing does not require blinding as the effect of genetic variants on disease outcome is relative to the allele used as a reference.

Reporting for specific materials, systems and methods

We require information from authors about some types of materials, experimental systems and methods used in many studies. Here, indicate whether each material, system or method listed is relevant to your study. If you are not sure if a list item applies to your research, read the appropriate section before selecting a response.

Materials & experimental systems

n/a	Involved in the study
☒	☐ Antibodies
☒	☐ Eukaryotic cell lines
☒	☐ Palaeontology and archaeology
☒	☐ Animals and other organisms
☒	☐ Clinical data
☒	☐ Dual use research of concern
☒	☐ Plants

Methods

n/a	Involved in the study
☒	☐ ChIP-seq
☒	☐ Flow cytometry
☒	☐ MRI-based neuroimaging

Plants

Seed stocks

Report on the source of all seed stocks or other plant material used. If applicable, state the seed stock centre and catalogue number. If plant specimens were collected from the field, describe the collection location, date and sampling procedures.

Novel plant genotypes

Describe the methods by which all novel plant genotypes were produced. This includes those generated by transgenic approaches, gene editing, chemical/radiation-based mutagenesis and hybridization. For transgenic lines, describe the transformation method, the number of independent lines analyzed and the generation upon which experiments were performed. For gene-edited lines, describe the editor used, the endogenous sequence targeted for editing, the targeting guide RNA sequence (if applicable) and how the editor was applied.

Authentication

Describe any authentication procedures for each seed stock used or novel genotype generated. Describe any experiments used to assess the effect of a mutation and, where applicable, how potential secondary effects (e.g. second site T-DNA insertions, mosiacism, off-target gene editing) were examined.
