# Supplementary material for: Nanoparticle-enriched mass spectrometry proteomics in British South Asians identifies links between genetic variants, plasma protein levels and disease risk
Source: Nat Genet. 2026 Jul 24;58(8):1866–76. doi: 10.1038/s41588-026-02697-6 (PMC13447103; doi:10.1038/s41588-026-02697-6)
Supplement: Supplementary file 3 [file 41588_2026_2697_MOESM3_ESM.pdf]

nature portfolio

Peer Review File

Nanoparticle enriched mass spectrometry proteomics in British South Asian individuals identifies mechanistic links between genetic variants, plasma protein levels and disease risk

Corresponding Author: Professor Maik Pietzner

Version 0:

Reviewer comments:

Reviewer #1

(Remarks to the Author)
Pietzner et. al. leveraged a nanoparticle coupled MS-based plasma proteomics profiling platform to quantify 8,079 circulating proteins in 1,464 individuals from the G&H cohort (of British Bangladeshi and Pakistani ancestry) and compared these results to 2 affinity-based platforms commonly used for proteomics. They demonstrated that utilization of this different proteomics quantification approach in this population with unique genetic makeup facilitates the identification of a large number of novel circulating proteins, pQTLs, and potential protein effectors of disease. This is a topic of interest to the scientific community (particularly given the inability of present high throughput techniques…for example to distinguish PTMs) and the authors should be commended for the effort and large number of analyses that have been conducted with rigor. However, there are results/conclusions that would benefit from much additional clarification as detailed below:

1. The reviewer appreciates the large effort already expended, but there is no distinct validation cohort for the new SEER data. The complementary information with the other two platforms is supportive for some findings, but doesn't exactly fulfill that goal.

2. Given possible ambiguity in protein group identification with MS based methods, did the authors conduct principal component analyses or correlation studies to quantify how strongly correlated to each other the Seer proteomic groups are?

3. How/what data was used to determine there was little evidence that protein isoform-specific targeting, unspecific targeting of affinity reagents, etc. (page 6, line 147) did not explain the low correlation between platforms? This is a major point without evidence.

4. No orthogonal technical validation in terms of top novel findings proteins identified by SEER. A subset of top findings should be validated by ELISA, targeted MS, etc.

5. Utilizing peptide and not protein group-level MS improved correlation in plasma levels improved correlations between MS and affinity-based measurements, but the peptides were only measured in less than half of the population (Page 6, line 142). Was this also true for the associated Olink and SomaLogic protein measurements?

6. What is the distribution of pleiotropic pQTLs seen with proteomic measurements using SOMA and Olink, and is this similar/greater/smaller than what was found in the MS pQTLs as a possible proxy measurement of platform specificity?

7. Of the 41 proteins with cis-pQTLs that are predicted to be destabilizing (page 9, line 232), how many of these were measured by the affinity-based methods, and what was the correlation in measurements to MS? Is there any direct evidence that these are destabilizing? This is an important issue.

8. The IGLV3-21 association with Graves' disease is intriguing but specultive, do the authors have any hypothesis on why there is missingness of gene expression in the intermediate B-cell? Additionally, is this lead cis-qQTL associations with circulating TRAb in larger databases? Most importantly, have they measured the IGLV3 protein in Graves' patients – this is critical, otherwise the entire mechanism/clinical implication is purely based on in silico analyses.

Reviewer #2

(Remarks to the Author)
This manuscript presents the largest and most comprehensive study conducted to date using nanoparticle enriched mass spectrometry on plasma proteomic samples from British participants of South Asian ancestry. The authors applied the Seer Proteograph XT platform (capturing 8,067 protein groups) alongside two affinity-based platforms - Somalogic 11k (capturing 9,685 proteins) and Olink HT (capturing 5,416 proteins) across over 1,400 samples in the Genes and Health cohort. They combine these proteomic data with comprehensive genetic data to reveal the following key insights: (1) The overall agreement between platforms for measurements of overlapping proteins is poor. This is aligned with other comparative investigations of proteomics technologies in White European cohorts; however, the authors go beyond past studies by highlighting how the poor agreement is likely driven by technical factors, with approximately 40% of Olink or Somalogic-based measures unlikely to produce concordant results; (2) The combined application of multiple proteomics technologies across ancestrally under-represented populations can power discovery of unique protein quantitative trait loci (pQTLs), here, the authors describe over 600 novel pQTLs not found in studies of White populations over 30 times the size of the present investigation; (3) Several novel pQTLs can be used to identify novel links between proteins and disease processes; here, the authors implicate 21 different proteins in the pathologies of 44 different illnesses, providing the exemplar or IGLV3-21 an Graves' disease. However, the authors note that such links between proteins and diseases can only be robustly established via very broad coverage of both the human proteome and the human phenome - integrating multiple lines of evidence from biobanks, genetic association catalogs, and orthogonal protein measurement techniques to minimize false negatives and optimize sensitivity.

This manuscript is another tour-de-force led by several pre-eminent experts in proteomics, genetics and population health. The authors should be commended on multiple fronts for the quality of the study - from the sophisticated design of Genes and Health itself, to the study's integration of both rare and common variants for proteogenomic target discovery, to the unprecedented biological breadth of the study - capturing well upwards of 10,000 unique proteins, to the timely and much-needed evaluation of several prominent proteomics technologies. Once published, this will become a landmark paper for the molecular epidemiology community, guiding future decision-making for population proteomics studies and proteogenomic target discovery initiatives. It is of very high relevance to the readership of Nature Genetics and I enthusiastically endorse its acceptance for publication following several (mostly modest) updates, outlined below:

1. The authors highlight how they have discovered more than 600 new pQTLs not reported in studies thirty times the size of this one, emphasizing the benefits of combining protein measures from multiple platforms in less widely studies ancestral populations. Have the authors considered conducting a sensitivity analysis to determine the potential cis- and trans-pQTL yields at even greater sample sizes? For example, Figures 2e and 2g of Sun et al. (https://www.nature.com/articles/s41586-023-06592-6/figures/2) highlight projected pQTL detection rates with increasing sample sizes and numbers of proteins using data from mostly White European individuals. It would be fascinating and quite informative for the broader community if a similar sensitivity analysis was performed in this British South Asian cohort.
2. The clinical translational potential of the findings is alluded to throughout the manuscript. Although the link between IGLV3-21 and Graves' disease is striking, the therapeutic implications are currently speculative; a note emphasizing the need for functional follow-up of this finding in the limitations paragraph is required. The authors note over 44 additional robust disease-protein associations in the manuscript, but most of these are buried in the Supplement and not expanded upon in the main text. If there are equally striking examples to IGLV3-21 and Graves' disease, I would strongly consider including one or two additional exemplars in the main text to better contextualize the translational utility of the proteogenomic analyses.
3. It is now quite commonplace for pQTL associations to be integrated into interactive online browsers for easy perusal of results. Do the authors plan to construct such a browser prior to publication? Building such a tool would further increase the impact of the manuscript. If the authors do not plan to do so, a brief justification would be helpful.
4. Have the authors assessed whether nanoparticle enrichment biases towards particular classes of proteins based on size and/or hydrophobicity, and were pre-analytical drivers of bias (as described by Korff et al: https://www.embopress.org/doi/full/10.1038/s44321-025-00309-0) evaluated as part of the present analysis?
5. Certain figures are dense and could benefit from clearer visualization for a general audience; this is particularly apparent for Figure 1 and Figure 5, where text fonts are tiny and titles are sometimes squashed together, making initial interpretation of the images more challenging.
6. Although the manuscript is extremely well-written, I noticed some typos or sections that could be updated for clarity. This includes:
a. Lines 68-70: "These communities" is plural, "despite its higher frequency…" is singular - please update for consistency, e.g., "despite their higher frequency"
b. Line 306: "proposed to be invalid" - proposed by whom? I assume that this is proposed by Reference #37 but it is not clear in the text.
c. Line 316: "i.e., that the altered protein sequence may infer…" Do the authors mean "interfere" rather than "infer"?
d. Line 387: "Our observations align with previous studies at this locus that has been reported…" - consider changing to, "our observations align with previous associations at this locus that have been reported…"
Lines 522 - 531: The language here reads somewhat informal compared to the rest of the manuscript. Consider updating sentences like, "An endeavor complicated by the fact, that many genetic variants likely regulate the expression of multiple genes in the proximity", to "This endeavor is complicated by the fact that many genetic variants likely regulate the expression of multiple proximal genes".

Reviewer #3

(Remarks to the Author)
Thanks for the opportunity to review this manuscript. The work is important to the field but I do have some comments and questions. The most important one is the 1st.

General comments

1. There are several sections in the manuscript where I think it would be important to con-sider also the sensitivity of the assays used and compared. Both affinity and MS prote-omics platforms are largely relative measurements and it can be challenging to distin-guish noise from signal. For example, comparisons of pQTL and direct correlation across platforms will be dependent on assay sensitivity and influence some of the key messages of the manuscript.
a. Please present details on detectability for each platform plus QC measures for removal of proteins with low detectability.
b. Also, the use of imputation for proteins with up to 30 % missingness for GWAS seems like quite a controversial decision. Was imputation used also for Olink and Somascan, as I assume there was missingness for those measurements too.
c. Were imputed values used also for assessment of correlation across platforms?
d. Please include a section in the discussion around detectability / limit of detection and how that may influence the conclusions of this work.
2. Please do a careful review of the language. Whilst it is largely grammatically correct, it reads as rather clunky and imprecise in some sections.
3. Also on language, the abstract has been written in present form, which looks a bit funny since the study has already been conducted.
4. Is there a description of the study population (the subset that was used here, not the whole cohort) that include e.g. age, sex, BMI, smoking etc? Apologies if I missed it.

Specific comments
Line 20: Suggest to change “underlying” with “associated with” -underlying implies an active pro-cess
Line 22-23 Suggest to change “integrate exome sequencing 23 and common non-coding varia-tion” to “here we aimed to identify protein quantitative trait loci using”, mainly since the frequency of the variant is not very important, it introduces the pQTL terminology early and since “integrate” is an obscure term.
Line 29: Were 1,200 variants or loci discovered? 1,200 variants seems low .
Line 30: provide basic statistical thresholds for the pQTL analysis
Line 33: Please change “integrate” to something more precise.
Line 34: With “consolidate”, do you mean “suggest”
Line 37: What is meant by “diverse” here? The study was conducted in South-Asians, which is a strength compared to previous literature but the present study did not include many different ethnic groups.
Line 47: “Protein targets” is not commonly used, and “preselected” is not the best way to describe the process of designing affinity panels since both Somascan and Olink have screened many more proteins during assay design. Better to say “5,000 targeted proteins”?
Line 54-56: I read this sentence several times and cannot make sense of it. Please review.
Line 59-63: Please review this sentence since it sounds a bit like a sales pitch. There are other types of MS enrichment methods that are also capable of reproducibly measure proteins, nano-particle-enrichment being one of them.
Line 64: Please avoid branding work “as the most comprehensive so far” and similar, not all litera-ture have been reviewed in the present manuscript, and some other MS studies have also includ-ed longitudinal samples.
Line 80: “protein groups” have not previously been introduced, please explain. If this means “sev-eral peptides for the same protein” I would suggest using simply “protein” to avoid confusion.
Line 90: Please explain why it is notable that only a smaller percentage of predicted secreted pro-teins were seen in the analyses? The so called “secretome” are mainly membrane and extracellu-lar proteins whereas many proteins observed in blood are leaked from cells. For this data to be interesting, I think there is a need to also include some abundance threshold. Did for example the proportion of predicted secreted proteins increase with more abundant proteins?
Line 241: It is stated that “We further identified evidence for 651 pQTLs that those may act via altered gene expression in one or more tissues”. Is it more relevant to assess overlap using cred-ible sets compared to formal coloc analysis or why was this method chosen? eQTL are known to regulate more than one gene and thus be less specific compared to pQTL. Also, I am not sure it is semantically correct to write “identified evidence” -it sounds more like a forensic investigation than research.
Line 252, figure 2d and continued on line 258: Are the examples illustrating a subset of the 1217 independent pQTL i.e. they were also detected using a linear model, or did the authors run ge-nome-wide analyses using dominant and recessive models?
I am not convinced that the plots show non-additive effects that would not be picked up in a line-ar model.
Line 283: Not sure if the lack of replication was “explained” by poor correlation or simply coincid-ed with proteins that were not closely correlated.
Line 291: The data on reciprocal replication of pQTL is valuable. However, another key reason for why affinity-based pQTL did not replicate using MS may be detectability. Please present data on MS detection limit for proteins with non-replicating pQTL.
Line 303: I agree that the authors have shown that lack of replication correlates well with how well the proteins correlate across platforms. This is in fact what you would expect. However, to what extent does low detectability explain the poor correlation? Please look into what the noise levels are for each respective platform (limit of detection), stratify by relative abundance and pre-sent replication proportion based on perhaps 3-4 categories of relative abundance.
Line 328: Title: The proteome is also a phenome. I would suggest to change the title to non-proteomic phenotypes or genetic traits

Line 329: When you write “identified pQTLs” does that include only the not previously reported ones or the novel?
Line 341: Considering that the NINJ1 trans-pQTL showed no other signals in GWAS, may it be related some artefact related to the MS quantification?
Line 241, 381 and other places: I suggest using pQTL for the plural form rather than pQTLs. Loci is already the plural form.
Line 349: Would it be possible to add trait information to figure 4? I understand the concept of the plot but think it would be very informative for the reader if some information on the associat-ed trait could be annotated.
Line 371: Colocalisation is not a model for causal inference per se so even if there is colocaliza-tion between disease risk (case-control GWAS) and protein levels, it does not necessarily mean that the protein mediates the disease risk since there is risk for horizonal pleiotropy that is not accounted for using coloc models. Have the authors considered running systematic MR as an alternative?
Line 392: I like the TMBIM1 example for improving prediction of an effector gene at this locus
Line 404: The CD40 example is nice but is not very new and the paragraph is rather thin on refer-ences to previous work on CD40, including MR/coloc work on CD40 protein levels. See for exam-ple PMID: 23696745, PMID: 36253349, PMID: 37563310, PMID: 33067605, PMID: 39935908 and many more.

Version 1:

Reviewer comments:

Reviewer #2

(Remarks to the Author)
The authors have effectively addressed my concerns. I have no further comments and commend them for what will represent an important contribution to the field.

Reviewer #3

(Remarks to the Author)
Apologies that I did not have time to review the updated version of this manuscript before the holidays. Overall, I think the manuscript has improved significantly since I saw it last and the new data on assay sensitivity increases interpretability of the findings. I do not have any new points to raise at this point.

Open Access This Peer Review File is licensed under a Creative Commons Attribution 4.0 International License, which permits use, sharing, adaptation, distribution and reproduction in any medium or format, as long as you give appropriate credit to the original author(s) and the source, provide a link to the Creative Commons license, and indicate if changes were made.
In cases where reviewers are anonymous, credit should be given to 'Anonymous Referee' and the source.
The images or other third party material in this Peer Review File are included in the article’s Creative Commons license, unless indicated otherwise in a credit line to the material. If material is not included in the article’s Creative Commons license and your intended use is not permitted by statutory regulation or exceeds the permitted use, you will need to obtain permission directly from the copyright holder.

To view a copy of this license, visit https://creativecommons.org/licenses/by/4.0/

Reviewers' Comments:

Reviewer #1 (Remarks to the Author):

Pietzner et. al. leveraged a nanoparticle coupled MS-based plasma proteomics profiling platform to quantify 8,079 circulating proteins in 1,464 individuals from the G&H cohort (of British Bangladeshi and Pakistani ancestry) and compared these results to 2 affinity-based platforms commonly used for proteomics. They demonstrated that utilization of this different proteomics quantification approach in this population with unique genetic makeup facilitates the identification of a large number of novel circulating proteins, pQTLs, and potential protein effectors of disease. This is a topic of interest to the scientific community (particularly given the inability of present high throughput techniques…for example to distinguish PTMs) and the authors should be commended for the effort and large number of analyses that have been conducted with rigor. However, there are results/conclusions that would benefit from much additional clarification as detailed below:

1. The reviewer appreciates the large effort already expended, but there is no distinct validation cohort for the new SEER data. The complementary information with the other two platforms is supportive for some findings, but doesn't exactly fulfill that goal.

R1 response 1 *We appreciate the comment and agree that external replication data from individuals of the same ancestry and measured with the same proteomic technology and coverage would be ideal and needed since we observed major obstacles in replicating pQTL signals even within our cohort. Since such opportunities do not exist, and to address the reviewer's comments, we have collated and now present several lines of evidence that directly and strongly support the validity of our presented pQTLs. Firstly, we note that almost half of all pQTLs (n=600) were previously identified, including measurements by orthogonal technologies (Supplementary Tab. 9). Secondly, for 38.7% of newly identified cis-pQTLs (n=239) that mapped to proteins not previously measured or not measured well before, we observed evidence that the same genetic variant also acted on expression of the corresponding gene in one or more tissues (Supplementary Tab. 9). Such results have previously been used and recommended to provide evidence for pQTL validation (e.g., Eldjarn et al 2023 Nature). Lastly, almost half (n=300) of newly identified pQTLs, or their proxies (r^2>0.6), have previously been reported in the GWAS Catalog for non-proteomic traits, additionally providing evidence for biological or clinical effects of the associated variants (Supplementary Tab. 9).*

We finally revised the limitation section to clearly state the need for replication of our findings in larger studies and other ancestral groups (p19, lines 539-544).

2. Given possible ambiguity in protein group identification with MS based methods, did the authors conduct principal component analyses or correlation studies to quantify how strongly correlated to each other the Seer proteomic groups are?

R1 response 2 *We followed the helpful suggestion from the reviewer and computed correlations among all measured protein groups. This revealed 28 clusters of highly correlated proteins groups (r^2>0.8) covering a total of 1544 protein groups, but those shared little to no sequence overlap that may lead to ambiguous peptide-to-protein group assignments. Apart*

from few examples hinting at the measurement of protein complexes rather than single proteins, the far largest cluster (n=1455 protein groups) was likely explained by cell type contamination during sample preparation. This conclusion is supported by the observation of similar clusters among the other platforms (e.g., n=448 proteins with SomaLogic), a marked enrichment of markers of blood cell lysis among the top cluster (odds ratio: 9.13; p-value = 1.1×10^{-17}), and the identification of distinct cis-pQTLs for some of the highly correlated protein groups within the largest cluster (n=49 cis-pQTLs).

We have added this analysis to the revised manuscript to also address concerns raised by reviewer 2, since they provide important insights into the comparative sensitivity to sample handling across proteomic technologies (p7, lines 160-171).

3. How/what data was used to determine there was little evidence that protein isoform-specific targeting, unspecific targeting of affinity reagents, etc. (page 6, line 147) did not explain the low correlation between platforms? This is a major point without evidence.

R1 response 3 *We apologize for the missing explanation and carefully revised the section to clearly outline the criteria used to identify potentially isoform targeting or missing specificity of reagents (p6/7, lines 145-159). Briefly, these analyses are based on performing correlation analysis using peptides instead of aggregated protein groups, matching for each protein targeted by affinity reagents all possible matching peptides using all amino acid sequences recorded for the respective proteins. We reasoned that affinity reagents that target a specific isoform of a given protein should best correlated with peptides specific to an isoform. Similar reasoning was behind unspecific targeting, i.e., the best correlating peptide is one shared by multiple proteins, and ambiguities in protein grouping, i.e., the peptide correlating best with the affinity reagent has not been used during protein group aggregation but was rather assigned to a different protein group not closely matching the affinity reagent.*

4. No orthogonal technical validation in terms of top novel findings proteins identified by SEER. A subset of top findings should be validated by ELISA, targeted MS, etc.

R1 response 4 *We acknowledge that targeted, better clinically approved, assays would further strengthen our findings, but admit that the required investment of precious sample material, staff time, and funding is beyond our current capabilities having already invested into three distinct proteomic technologies to corroborate findings. We now provide orthogonal technical validation for 18 protein targets, for which corresponding clinical measures were available based on harmonized electronic health records. We observed significant associations for 14 out 18 examples (Supplementary Tab. 18) despite substantial differences between measurement timepoints. This included, for example, strong associations with Immunoglobulin A, G, and M measurements using approved clinical assays (e.g., 183 individuals with IgA measurements: beta=0.74; $p<5.1 \times 10^{-28}$) that support the capability of our mass spectrometry approach to capture immunological signals.*

We further provide several lines of external replication for findings we particularly highlight that improved credibility of the identified association. For example, we replicated 71.6% (48 out of 67 among well correlated, r>0.5, protein measures) of novel pQTLs obtained with Seer using two alternative technologies that use completely distinct modes of measuring the

protein target. We further observed that among the newly identified cis-pQTLs, 21 could immediately be linked genetically to metabolic reactions and pathways they are involved in. For example, the cis-pQTL (chr12:120738280:G>A) for short-chain specific acyl-CoA dehydrogenase (ACADS) that has been previously reported to associated with plasma levels of short chain fatty acids. However, we appreciate that such efforts are needed to provide context, but do not provide highest analytical evidence and expanded the limitation section accordingly (p19, lines 543-544).

5. Utilizing peptide and not protein group-level MS improved correlation in plasma levels improved correlations between MS and affinity-based measurements, but the peptides were only measured in less than half of the population (Page 6, line 142). Was this also true for the associated Olink and SomaLogic protein measurements?

R1 response 5 *We apologise for the lack of information. By the nature of the SomaLogic and Olink assays, measurements were available for the entire cohort and the reduction in the study population was due to the availability of peptide measurements. We clarified this section in the revised version of the manuscript (p6, lines 152-153).*

6. What is the distribution of pleiotropic pQTLs seen with proteomic measurements using SOMA and Olink, and is this similar/greater/smaller than what was found in the MS pQTLs as a possible proxy measurement of platform specificity?

R1 response 6 *Restricting to sets of proteins common to either platform comparison, we observed a similar fraction of pleiotropic pQTLs (≥5 associated proteins) for Olink and MS (n=4 vs n=6; across 1891 targets) while SomaLogic had a much higher number of pleiotropic trans-pQTLs (n=43 vs n=8 in MS across 3404 protein targets). The latter aligns with our present and previous observation, that a set of pleiotropic pQTLs seems to interfere with measurement properties of the SomaLogic but no other platforms, including missense variation within CFH that increased DNA binding affinity of the complement factor H encoded at the locus (p11, lines 300-304).*

7. Of the 41 proteins with cis-pQTLs that are predicted to be destabilizing (page 9, line 232), how many of these were measured by the affinity-based methods, and what was the correlation in measurements to MS? Is there any direct evidence that these are destabilizing? This is an important issue.

R1 response 7 *Half of the 28 cis-pQTL with such evidence and available measures on either affinity platform replicated (directionally concordant and $p<10^{-5}$), with those not replicating being poorly correlated across technologies (median correlations: 0.60 versus 0.20). We note that all protein measurements linked to these variants were derived excluding the peptide of potential concern, and unaffected peptides showed strong support of an association with plasma abundance. Our plasma level measurements therefore supported stability predictions from in silico models, although we acknowledge that experimental work would be needed to provide further evidence for protein destabilizing effects of these variants to possibly explain shorter half-lives or diminished secretion/leakage into blood.*

8. The IGLV3-21 association with Graves' disease is intriguing but specultive, do the authors have any hypothesis on why there is missingness of gene expression in the intermediate B-cell? Additionally, is this lead cis-qQTL associations with circulating TRAb in larger databases? Most importantly, have they measured the IGLV3 protein in Graves' patients – this is critical, otherwise the entire mechanism/clinical implication is purely based on in silico analyses.

R1 response 8 *Thank you for your interest in this intriguing example, that we highlight. To address the reviewer's questions and comments, we now provide more details and clarify that our findings should be considered as hypothesis (p16, lines 441-462).*
Regarding intermediate B cells, those were found only in small proportions in the original subset of the TenK10K study, so missing expression is most likely a result of low cell counts rather than truly missing expression of these genes in relevant cell types. We further provide evidence that the cis-pQTL associates with lower TRAb titers in the independent FinnGen cohort (beta=-0.08; p-value<$5.2x10^{-4}$) (p16, lines 441-446). Most importantly, we now clarify that the collective evidence points to the individual composition of TRAbs rather than IGLV3-21 being a novel strong biomarker for Graves' disease.

Reviewer #2 (Remarks to the Author):

This manuscript presents the largest and most comprehensive study conducted to date using nanoparticle enriched mass spectrometry on plasma proteomic samples from British participants of South Asian ancestry. The authors applied the Seer Proteograph XT platform (capturing 8,067 protein groups) alongside two affinity-based platforms - Somalogic 11k (capturing 9,685 proteins) and Olink HT (capturing 5,416 proteins) across over 1,400 samples in the Genes and Health cohort. They combine these proteomic data with comprehensive genetic data to reveal the following key insights: (1) The overall agreement between platforms for measurements of overlapping proteins is poor. This is aligned with other comparative investigations of proteomics technologies in White European cohorts; however, the authors go beyond past studies by highlighting how the poor agreement is likely driven by technical factors, with approximately 40% of Olink or Somalogic-based measures unlikely to produce concordant results; (2) The combined application of multiple proteomics technologies across ancestrally under-represented populations can power discovery of unique protein quantitative trait loci (pQTLs), here, the authors describe over 600 novel pQTLs not found in studies of White populations over 30 times the size of the present investigation; (3) Several novel pQTLs can be used to identify novel links between proteins and disease processes; here, the authors implicate 21 different proteins in the pathologies of 44 different illnesses, providing the exemplar or IGLV3-21 an Graves' disease. However, the authors note that such links between proteins and diseases can only be robustly established via very broad coverage of both the human proteome and the human phenome - integrating multiple lines of evidence from biobanks, genetic association catalogs, and orthogonal protein measurement techniques to minimize false negatives and optimize sensitivity.

This manuscript is another tour-de-force led by several pre-eminent experts in proteomics, genetics and population health. The authors should be commended on multiple fronts for the quality of the study - from the sophisticated design of Genes and Health itself, to the study's integration of both rare and common variants for proteogenomic target discovery, to the unprecedented biological breadth of the study - capturing well upwards of 10,000 unique

proteins, to the timely and much-needed evaluation of several prominent proteomics technologies. Once published, this will become a landmark paper for the molecular epidemiology community, guiding future decision-making for population proteomics studies and proteogenomic target discovery initiatives. It is of very high relevance to the readership of Nature Genetics and I enthusiastically endorse its acceptance for publication following several (mostly modest) updates, outlined below:

1. The authors highlight how they have discovered more than 600 new pQTLs not reported in studies thirty times the size of this one, emphasizing the benefits of combining protein measures from multiple platforms in less widely studies ancestral populations. Have the authors considered conducting a sensitivity analysis to determine the potential cis- and trans-pQTL yields at even greater sample sizes? For example, Figures 2e and 2g of Sun et al. (https://www.nature.com/articles/s41586-023-06592-6/figures/2) highlight projected pQTL detection rates with increasing sample sizes and numbers of proteins using data from mostly White European individuals. It would be fascinating and quite informative for the broader community if a similar sensitivity analysis was performed in this British South Asian cohort.

R2 response 1 *We agree with the reviewer on the value of such an analysis. Unfortunately, the comparatively small sample size of our study compared to UK Biobank (~1.5k vs 50k) did not allow us deriving meaningful estimates. For example, the work by Sun et al did consider sample sizes of at least 5000 to derive meaningful estimates to forecast gain in associations with increasing sample size. We note that the 1217 identified associations identified in our study represents an approximately linear extrapolation to the estimates presented by Sun et al for the smaller Non-European sets within UK Biobank.*

2. The clinical translational potential of the findings is alluded to throughout the manuscript. Although the link between IGLV3-21 and Graves' disease is striking, the therapeutic implications are currently speculative; a note emphasizing the need for functional follow-up of this finding in the limitations paragraph is required. The authors note over 44 additional robust disease-protein associations in the manuscript, but most of these are buried in the Supplement and not expanded upon in the main text. If there are equally striking examples to IGLV3-21 and Graves' disease, I would strongly consider including one or two additional exemplars in the main text to better contextualize the translational utility of the proteogenomic analyses.

R2 response 2 *We agree with the reviewer that possible implications of the IGLV3-21 and Grave's disease finding are speculative and rephrased the entire section carefully to outline that additional work is needed before any therapeutic claim might be made (p16, lines 48-449). We further expanded upon examples to demonstrate potentially translation value of our study putting emphasize on the distinctive capabilities of mass spectrometry-based platforms (p14/15, lines 395-419). Briefly, we exemplify how peptide level association confirmed exon skipping as likely underlying mechanism for an effect of ERO1B on type 2 diabetes and newly demonstrate a protective rather than adverse effect that emphasizes a crucial role of ERO1-like protein beta (ERO1B) in fine-tunning oxidative environments in pancreatic beta cells to balance endoplasmic reticulum stress with biogenesis of insulin. We further provide evidence for a putative protective role for DNASE1L1 in type 2 diabetes.*

3. It is now quite commonplace for pQTL associations to be integrated into interactive online browsers for easy perusal of results. Do the authors plan to construct such a browser prior to publication? Building such a tool would further increase the impact of the manuscript. If the authors do not plan to do so, a brief justification would be helpful.

R2 response 3 *We appreciate the reviewer's suggestion and agree that interactive pQTL browsers are valuable resources. At this stage, however, we do not plan to develop a dedicated web interface for this dataset. Our decision is based on two considerations. First, creating and maintaining a robust, user-friendly browser requires substantial long-term infrastructural and personnel support, which goes beyond the scope and resources of the present study. This is of particularly relevance, since the current effort is of comparatively small size restricting the usefulness of pQTL look-ups and we provide rigorous fine-mapping results in the Supplementary tables for downstream MR-type applications. Second, the full GWAS summary statistics and accompanying code will be openly released upon publication, enabling the community to integrate these results into existing platforms or bespoke tools as needed.*

We believe this approach ensures transparency and broad reusability, while allowing us to focus resources on delivering high-quality analyses and documentation.

4. Have the authors assessed whether nanoparticle enrichment biases towards particular classes of proteins based on size and/or hydrophobicity, and were pre-analytical drivers of bias (as described by Korff et al: https://www.embopress.org/doi/full/10.1038/s44321-025-00309-0) evaluated as part of the present analysis?

R2 response 4 *We have followed the helpful suggestions of the reviewer and now show that physical properties of proteins explain to some extent differential protein coverage across technologies (**Extended Data Fig. 2**; p5, lines 106-121). Briefly, we observed that proteins high in aspartate, but low in cysteine and serine are enriched in the MS-based workflow, which may relate to how proteins bind to and are subsequently liberated from nanoparticles for subsequent enzymatic digestion. The list of discriminative features was thereby shared with those selected to characterise 4580 proteins curated to be present in plasma across hundreds of studies (Geyer et al. 2024* J. Proteome Res.*). In contrast, both affinity-based technologies showed a tendency to capture proteins with high percentages of polar amino acids that are frequently enriched on the surface of proteins to form hydrophilic sites that may make good epitopes. Length and size of proteins was not selected by our machine learning algorithm to explain coverage and the contrast to the results by Korff et al. might be best explained by asking a related but different question. That is, not only comparing within mass spectrometry workflows but beyond on what type of proteins may be missed by MS.*

*We further investigated potential sources of pre-analytical biases, including sample handling artefacts, in detail before conducting genetic work. Connecting principal component analysis with available blood counts thereby provided evidence that some of those may reflect such bias. However, systematically testing adjustment strategies that utilized proteomic PCs in genetic analysis revealed an increase in association statistics for pleiotropic trans-pQTL without improving cis-pQTL discovery. Since we report effects genome-wide, we decided to not include protein PCs in our genetic analysis to avoid inflation of uncertain findings in trans. Further, as part of the review (see **R1 response 2**), we now observe a large cluster of proteins*

on the Seer and SomaLogic platforms that are likely indicative of sample handling being the sole source of their variation in plasma. We have added a brief description outlining those findings and how they may impact the presented findings (p7, lines 160-171). We note, that the collective observations point to possible false-negative rather than false-positive findings, that is, not impairing any of the presented conclusions on genetic determinants and inferred clinical examples.

5. Certain figures are dense and could benefit from clearer visualization for a general audience; this is particularly apparent for Figure 1 and Figure 5, where text fonts are tiny and titles are sometimes squashed together, making initial interpretation of the images more challenging.

R2 response 5 *We carefully revised all figures to improve visualisation.*

6. Although the manuscript is extremely well-written, I noticed some typos or sections that could be updated for clarity. This includes:
a. Lines 68-70: "These communities" is plural, "despite its higher frequency…" is singular - please update for consistency, e.g., "despite their higher frequency"
b. Line 306: "proposed to be invalid" - proposed by whom? I assume that this is proposed by Reference #37 but it is not clear in the text.
c. Line 316: "i.e., that the altered protein sequence may infer…" Do the authors mean "interfere" rather than "infer"?
d. Line 387: "Our observations align with previous studies at this locus that has been reported…" - consider changing to, "our observations align with previous associations at this locus that have been reported…"
Lines 522 - 531: The language here reads somewhat informal compared to the rest of the manuscript. Consider updating sentences like, "An endeavor complicated by the fact, that many genetic variants likely regulate the expression of multiple genes in the proximity", to "This endeavor is complicated by the fact that many genetic variants likely regulate the expression of multiple proximal genes".

R2 response 6 *We thank the reviewer for the careful read of our manuscript and corrected the typos accordingly or rephrased sections to improve clarity.*

Reviewer #3 (Remarks to the Author):

Thanks for the opportunity to review this manuscript. The work is important to the field but I do have some comments and questions. The most important one is the 1st.

General comments

1. There are several sections in the manuscript where I think it would be important to con-sider also the sensitivity of the assays used and compared. Both affinity and MS prote-omics platforms are largely relative measurements and it can be challenging to distin-guish noise from signal. For example, comparisons of pQTL and direct correlation across platforms will be dependent on assay sensitivity and influence some of the key messages of the manuscript.

R3 response 1 *We agree with the reviewer and our in-depth analysis of factors contributing to poor correspondence across platforms clearly highlighted detectability and missing values as one important source. We now provide detailed subgroup analysis, including assay correspondence but also pQTL replication/discovery, and highlight the relevance of detectability in the revised version of the manuscript in multiple sections (e.g., p5/6, lines 126-133 or p11 lines 289-292).*

a. Please present details on detectability for each platform plus QC measures for removal of proteins with low detectability.

R3 response 1a *We provide extensive data on detectability in Supplementary Tables 1-3 for each protein captured. We did not remove proteins due to missingness or below LOD values, to provide a fair comparison across platforms. We have now added correlation and pQTL replication analysis stratified by estimated LOD levels, which outlines the importance of considering assay sensitivity. However, we note that, when comparing to the SomaLogic platform, below LOD estimation did not explain bimodal distributions. These results have been incorporated in the revised manuscript (e.g., p5/6, lines 126-133) and updated Supplementary Material (e.g., Supplementary Tab. 13).*

b. Also, the use of imputation for proteins with up to 30 % missingness for GWAS seems like quite a controversial decision. Was imputation used also for Olink and Somascan, as I assume there was missingness for those measurements too.

R3 response 1b *We agree with the reviewer that imputation is always a concern and was done here to achieve computational efficiency when running billions of association tests in parallel. To filter any potentially spurious findings, we used not imputed peptide level data that provided support for almost all findings (1026 out of 1217) indicating no inflation of false-positive findings due to imputation. No imputation was done for SomaLogic or Olink, since missingness was low as vendors provided values even if assays were below a putative LOD (the computation of which is not straightforward due to the nature of both assays). We note that including such very low values has proven important in proteogenomic studies as we and others observed inflation of false-positive findings otherwise, in particular for rare variants.*

c. Were imputed values used also for assessment of correlation across platforms?

R3 response 1c *No imputation was done prior correlation analyses, and we clarified this in the revised manuscript (p30, lines 851-852).*

d. Please include a section in the discussion around detectability / limit of detection and how that may influence the conclusions of this work.

R3 response 1d *We followed the helpful suggestion from the reviewer and expanded upon the relevance of detectability to explain some of the discordances observed across proteomic platforms (p18, lines 49-497).*

2. Please do a careful review of the language. Whilst it is largely grammatically correct, it reads as rather clunky and imprecise in some sections.

R3 response 2 *We apologise for the imprecise language and carefully reviewed the entire manuscript.*

3. Also on language, the abstract has been written in present form, which looks a bit funny since the study has already been conducted.

R3 response 3 *We understand the confusion, but this type of format is now requested by Nature Genetics.*

4. Is there a description of the study population (the subset that was used here, not the whole cohort) that include e.g. age, sex, BMI, smoking etc? Apologies if I missed it.

R3 response 4 *We apologize for the missing information on study characteristics beyond age and sex, that has now been added to the revised manuscript (p4, lines 76-77).*

Specific comments
Line 20: Suggest to change "underlying" with "associated with" -underlying implies an active pro-cess

R3 response 5 *This sentence is no longer part of the revised abstract to adhere to journal guidelines for the length of the abstract.*

Line 22-23 Suggest to change "integrate exome sequencing 23 and common non-coding varia-tion" to "here we aimed to identify protein quantitative trait loci using", mainly since the frequency of the variant is not very important, it introduces the pQTL terminology early and since "integrate" is an obscure term.

R3 response 6 *The sentence has been deleted from the rephrased abstract and replaced with a more generic statement(p2, lines 25-26).*

Line 29: Were 1,200 variants or loci discovered? 1,200 variants seems low .

R3 response 7 *We clarified in the revised version of the manuscript, that 1217 locus – protein group associations have been identified (p2, lines 25-26).*

Line 30: provide basic statistical thresholds for the pQTL analysis

R3 response 8 *We added the corrected p-value to the abstract (p2, lines 25-26).*

Line 33: Please change "integrate" to something more precise.

R3 response 9 *We rephrased the sentence to be more precise (p2, line 28).*

Line 34: With "consolidate", do you mean "suggest"

R3 response 10 *We changed 'consolidate' to 'suggest', given the speculative nature of the example.*

Line 37: What is meant by "diverse" here? The study was conducted in South-Asians, which is a strength compared to previous literature but the present study did not include many different ethnic groups.

R3 response 11 *The reviewer is right. Our study did not include other ancestries and we carefully revised any reference to 'diverse' throughout the manuscript.*

Line 47: "Protein targets" is not commonly used, and "preselected" is not the best way to describe the process of designing affinity panels since both Somascan and Olink have screened many more proteins during assay design. Better to say "5,000 targeted proteins"?

R3 response 12 *We thank the reviewer for providing guidance on this important but hard to convey manner. We adopted the proposal in the revised version of the manuscript (p3, line 41)*

Line 54-56: I read this sentence several times and cannot make sense of it. Please review.

R3 response 13 *We rephrased the sentence to clarify the contribution and limitations of previous mass spectrometry-based pQTL efforts (p3, lines 48-50).*

Line 59-63: Please review this sentence since it sounds a bit like a sales pitch. There are other types of MS enrichment methods that are also capable of reproducibly measure proteins, nano-particle-enrichment being one of them.

R3 response 14 *We rephrased the sentence accordingly (p3, lines 54-56).*

Line 64: Please avoid branding work "as the most comprehensive so far" and similar, not all litera-ture have been reviewed in the present manuscript, and some other MS studies have also includ-ed longitudinal samples.

R3 response 15 *We acknowledge that our references are not exhaustive and never intended to diminish the work done by others. We toned down our wording accordingly (p3, line 58).*

Line 80: "protein groups" have not previously been introduced, please explain. If this means "sev-eral peptides for the same protein" I would suggest using simply "protein" to avoid confusion.

R3 response 16 *We rephrased the sentence to introduce the concept of protein groups, as we believe it is important to make that distinction more clearly given the ambiguities involved in mapping peptides to proteins (p4, lines 77-80). While this adds a level of complexity for the reader, it aligns with request by this and other reviewers to increase precision in language.*

Line 90: Please explain why it is notable that only a smaller percentage of predicted secreted pro-teins were seen in the analyses? The so called "secretome" are mainly membrane and

extracellu-lar proteins whereas many proteins observed in blood are leaked from cells. For this data to be interesting, I think there is a need to also include some abundance threshold. Did for example the proportion of predicted secreted proteins increase with more abundant proteins?

R3 response 17 *The relevance and origin of proteins that we can measure in blood is an ongoing debate and strongly limits our ability to translate findings into drugs or the clinics. All protein platforms measured proteins across the entire abundance spectrum (see figure below) and our decision to highlight proteins predicted to be secreted into blood was motivated by findings that those are enriched among proteins associated with a pQTL (e.g., Koprulu et al Nature Metabolism 2023).*

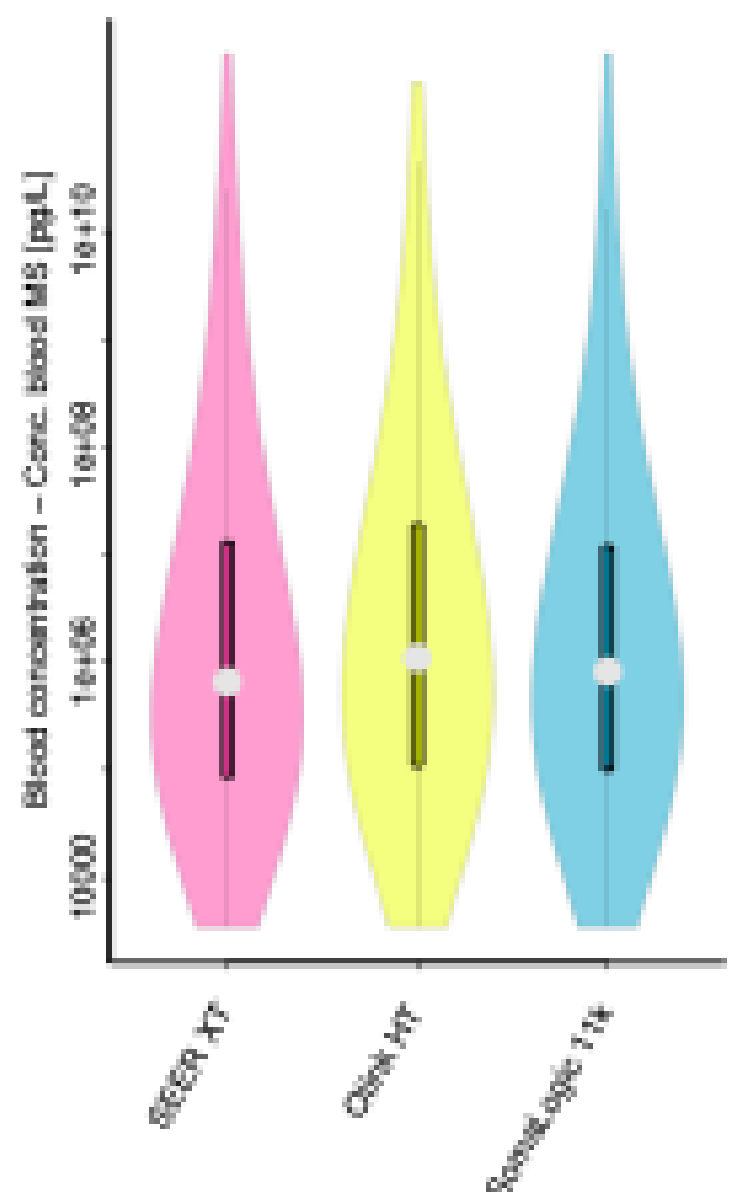

Figure *Distribution of protein abundance across technologies. Blood concentrations were obtained from the data curated at the human protein atlas based on hundreds of MS experiments.*

Line 241: It is stated that "We further identified evidence for 651 pQTLs that those may act via altered gene expression in one or more tissues". Is it more relevant to assess overlap using cred-ible sets compared to formal coloc analysis or why was this method chosen? eQTL are known to regulate more than one gene and thus be less specific compared to pQTL. Also, I am not sure it is semantically correct to write "identified evidence" -it sounds more like a forensic investigation than research.

R3 response 18 *Statistically speaking, assessing the overlap of credible sets is how colocalization works. However, colocalization assumes conserved LD-blocks across traits that are compared, and that is why we opted for comparing credible set overlap based on the results of ancestry-matched fine-mapping in our study and as done by the eQTL Catalog. Lead credible variants from our analysis with high posterior inclusion probabilities in the eQTL catalogue (PIP>0.1) thereby likely indicate colocalising signals. We restricted those analysis to cis-pQTLs and matching gene expression statistics for the protein coding gene to minimize effects from local pleiotropy. We further argue, that pQTLs may currently appear more specific than eQTLs given the limited coverage of proteins circulating in blood and the general ability*

of blood measurements to reflect tissue action. We rephrased the sentence to describe more clearly what had been done (p10, lines 254-256).

Line 252, figure 2d and continued on line 258: Are the examples illustrating a subset of the 1217 independent pQTL i.e. they were also detected using a linear model, or did the authors run ge-nome-wide analyses using dominant and recessive models?
I am not convinced that the plots show non-additive effects that would not be picked up in a line-ar model.

R3 response 19 *The reviewer is right, we restricted testing for non-additive effects to the identified 1217 pQTLs and did not rerun genome-wide association testing using different models of inheritance. This is based on our own but also the experiences from others in QTL studies, for which the massively increased computational burden and environmental costs did not reveal findings that have not already been detected using standard additive models. The analysis hence represents a refinement of the likely underlying genetic model.*

Line 283: Not sure if the lack of replication was "explained" by poor correlation or simply coincid-ed with proteins that were not closely correlated.

R3 response 20 *We rephrased the sentence for clarity (p11, lines 285-286).*

Line 291: The data on reciprocal replication of pQTL is valuable. However, another key reason for why affinity-based pQTL did not replicate using MS may be detectability. Please present data on MS detection limit for proteins with non-replicating pQTL.

R3 response 21 *We agree that detectability can mask replication, and now provide replication statistics by detectability bins (Supplementary Tab. 13). However, we observed little evidence that missingness on the MS platform accounted for the lack of replication of pQTLs discovered using affinity reagents, but rather poor alignment of measured values (Supplementary Tab. 12/13).*

Line 303: I agree that the authors have shown that lack of replication correlates well with how well the proteins correlate across platforms. This is in fact what you would expect. However, to what extent does low detectability explain the poor correlation? Please look into what the noise levels are for each respective platform (limit of detection), stratify by relative abundance and pre-sent replication proportion based on perhaps 3-4 categories of relative abundance.

R3 response 22 *We followed the helpful recommendation of the reviewer and now provide replication rates stratified by detection limits of the validation platform (Supplementary Tab. 12). Briefly, we observe a strong relation between detectability and replication when taking MS-discovered pQTLs forward to the Olink HT and to a far lesser extent the SomaLogic 11k platform. For example, two in three pQTLs replicated in the bin of highest detectability vs less than one in five in the bin of highest number of values below LOD (new Supplementary Tab. 12). This effect was less pronounced when comparing to the SomaLogic 11k platform due to very low values below a predicted LOD. We note that aiming for replication of affinity-based pQTLs using MS was less influenced by missingness, with values only marginally changing*

(e.g., 46.7% vs 30.1% Olink HT -> Seer XT). We revised the corresponding sections in the manuscript to clearly highlight these effects (p5/6, lines 126-133 or p11 lines 289-292).

Line 328: Title: The proteome is also a phenome. I would suggest to change the title to non-proteomic phenotypes or genetic traits

R3 response 23 *We agree with the reviewer that the 'phenome' is a rather unspecific term and changed the section header accordingly. It now reads 'Intersection of pQTLs with non-proteomics traits in the GWAS Catalog'.*

Line 329: When you write "identified pQTLs" does that include only the not previously reported ones or the novel?

R3 response 24 *We clarified that this sentence referred to all pQTLs passing statistical significance in our study irrespective of novelty, since it also provides a means to assess biological plausibility of findings (p12, line 325).*

Line 341: Considering that the NINJ1 trans-pQTL showed no other signals in GWAS, may it be related some artefact related to the MS quantification?

R3 response 25 *We investigated this possibility in more detail and observed no evidence for replication using affinity reagents, even for well correlating targets, supporting the suspicion of the reviewer. We have carefully revised the corresponding sections to suggest the possibility of a platform-specific finding (e.g., p12, line 338-340).*

Line 241, 381 and other places: I suggest using pQTL for the plural form rather than pQTLs. Loci is already the plural form.

R3 response 26 *We agree with the reviewer, that pQTL can be used as abbreviation for both, protein quantitative trait locus or loci. To align with the reporting in the field (e.g., Sun et al Nature 2023), we now introduce pQTL as 'protein quantitative trait locus' (p3, lines 38) and refer to pQTLs if more than one is meant.*

Line 349: Would it be possible to add trait information to figure 4? I understand the concept of the plot but think it would be very informative for the reader if some information on the associat-ed trait could be annotated.

R3 response 27 *We redesigned the figure to now indicate broad anatomical concepts to increase the information content.*

Line 371: Colocalisation is not a model for causal inference per se so even if there is colocalization between disease risk (case-control GWAS) and protein levels, it does not necessarily mean that the protein mediates the disease risk since there is risk for horizonal pleiotropy that is not accounted for using coloc models. Have the authors considered running systematic MR as an alternative?

R3 response 28 *We agree with the reviewer that colocalisation cannot infer causality, this is why we collated data across rare and common genetic variation, mouse models, and tissue expression patterns of proteins highlighted by colocalisation to increase confidence in putative causal association. This strategy builds on our and others body of work investigating suitability of coloc/MR to assess a potential causal role of proteins. Briefly, any cis-MR finding needs to be supported by colocalisation to rule out confounding by LD, doing colocalisation first therefore already ensures two relevant criteria for cis-MR: 1) relevance (the signal is strong for the exposure) and 2) no confounding by LD. We further refrained from implementing cis-MR more widely using credible set variants, since those may differ across ancestries and many coding variants may not be available in GWAS data largely based on imputed genotypes. Not at least, even systematic cis-MR findings cannot necessarily distinguish vertical from horizontal pleiotropy since they may still rely on a single instrument in most cases.*

Line 392: I like the TMBIM1 example for improving prediction of an effector gene at this locus

R3 response 29 *We thank the reviewer for the positive assessment of this example.*

Line 404: The CD40 example is nice but is not very new and the paragraph is rather thin on refer-ences to previous work on CD40, including MR/coloc work on CD40 protein levels. See for exam-ple PMID: 23696745, PMID: 36253349, PMID: 37563310, PMID: 33067605, PMID: 39935908 and many more.

R3 response 30 *We agree with the reviewer that plasma levels of CD40 have been reported to be associated with a wide range of diseases by us and others, including the beautiful early work by Li et al 2013 PLoS Genet, and we added references accordingly. However, the reason why we opted to highlight CD40 was justified by the level of information converging on this example and topical developments in clinical trials. The locus has been linked to GD pathology almost three decades ago (https://pubmed.ncbi.nlm.nih.gov/9837828/) but therapeutic modulation is still in its infancy, we hope that our work contributes to drug development acknowledging safety signals, such as for leukaemia's. It further serves as a contrast for the IGLV3-21 example, that showed much more disease specificity and hence highlights the rigour needed to provide tangible candidates for clinical translation.*
